# Thermally Driven Reorganization of Aurophilic AIE Aggregates in Microheterogeneous Acetonitrile/Water Media

**DOI:** 10.1021/acs.inorgchem.6c03111

**Published:** 2026-08-20

**Authors:** Ainhoa Rodríguez-Gobernado, Inés Soldevilla, Andrea Pinto, Isabel Esteban-Díez, Laura Rodríguez, Miguel Monge, María Rodríguez-Castillo, José M. López-de-Luzuriaga

**Affiliations:** † Departamento de Química, Instituto de Investigación en Química de la Universidad de La Rioja (IQUR), Universidad de La Rioja, Complejo Científico Tecnológico, Logroño 26006, Spain; ‡ Departament de Química Inorgànica i Orgànica, Secció de Química Inorgànica, Universitat de Barcelona, Martí i Franqués 1−11, Barcelona E-08028, Spain; § Institut de Nanociéncia i Nanotecnologia, Universitat de Barcelona, Barcelona 08028, Spain

## Abstract

We report a combined spectroscopic, kinetic, and computational study of aggregation-induced emission (AIE) in tetranuclear Au­(I)/Ag­(I) complexes in microheterogeneous acetonitrile/water mixtures. Time-resolved UV–vis spectroscopy and multivariate analysis reveal that once AIE aggregates are formed, a further aggregation process proceeds via a continuous, cooperative reorganization pathway rather than classical nucleation–growth. The process follows compressed-exponential kinetics and exhibits a well-defined activation barrier (*E*_a_ = 86 kJ·mol^–1^). Dynamic light scattering confirms a hierarchical growth from nanometric aggregates to micron-scale species leading to precipitation. DFT calculations show that interunit interactions (116 kJ·mol^–1^), dominated by aurophilic and π-stacking forces, govern aggregate stability and are mechanistically linked to the kinetic barrier arising from their partial reorganization. Conductor-like Screening Model (COSMO) analysis further indicates weak solvation, rationalizing the dominance of intermolecular interactions.

## Graphical abstract

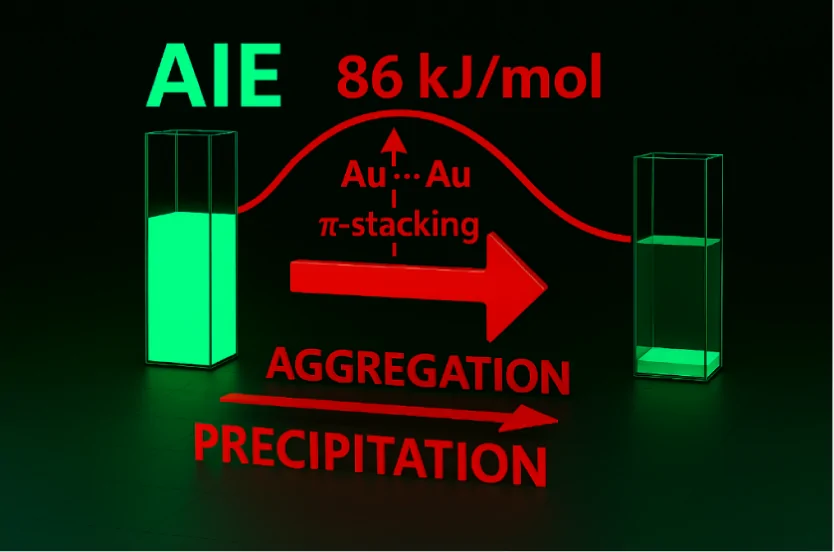

## Introduction

Aggregation-caused quenching has long represented a fundamental limitation for conventional luminophores, as molecular aggregation in concentrated solutions or solid states often enhances non radiative decay pathways through strong π-π stacking, excimer formation, and vibrational relaxation. In 2001, Tang and coworkers introduced the concept of aggregation induced emission (AIE), describing the counterintuitive phenomenon whereby non emissive or weakly emissive molecules in dilute solution become highly luminescent upon aggregation.^1^ Since this seminal discovery, AIE has developed into a broad and highly active research field encompassing organic molecules, polymers, coordination compounds and metal based luminogens, with applications ranging from optoelectronic devices and chemical sensors to bioimaging and stimuli responsive materials.^2–7^ The restriction of intramolecular motions (RIM), including suppression of internal rotations (RIR) and vibrations (RIV), is widely accepted as the dominant microscopic mechanism responsible for the enhancement of radiative decay in aggregated states.^2,3^

Among metal based AIE systems, closed shell d^10^ gold­(I) complexes occupy a distinguished position due to their ability to engage in short-range aurophilic interactions, typically manifested as Au···Au contacts in the 3.0–3.5 Å range.^8–10^ These metallophilic interactions not only impart structural rigidity to supramolecular assemblies but also generate electronically coupled excited states, such as metal centered or metal–metal to ligand charge transfer (MMLCT) states, that greatly enhance emission efficiency and frequently give rise to room temperature phosphorescence.^8,11^ As a result, a wide variety of mono, di, and heterometallic Au­(I)/Ag­(I) complexes have been reported to display AIE behavior in the solid state or in mixed water/organic solvent systems, where aurophilicity, π-π interactions, and solvophobic effects act cooperatively to drive aggregation.^11,12^

Mixed water-organic solvents, particularly water/acetonitrile systems, have become a widely employed platform to trigger aggregation induced emissions in gold­(I) complexes. Increasing water content progressively destabilizes molecular solvation and promotes supramolecular association, often yielding intense emission maxima at high water fractions.^11,12^ Importantly, water/acetonitrile mixtures are now recognized as intrinsically microheterogeneous media, in which transient solvent nanodomains, maintained by hydrogen bonded water networks and dipolar organic components, coexist over nanometric length scales.^13–15^ Such microheterogeneity has been shown to influence molecular solvation, diffusion, and excited state dynamics, suggesting that aggregation processes occurring in these media may deviate substantially from classical homogeneous kinetic models.

Despite the extensive photophysical characterization of AIE active gold­(I) aggregates, a key aspect remains largely unexplored: the kinetics and energetic landscape governing aggregate formation, reorganization, and destabilization. Most previous studies rely predominantly on steady state spectroscopy, lifetime measurements, or equilibrium temperature dependent emission trends, frequently ascribing observed effects to aggregation or disaggregation without resolving their temporal evolution. When kinetic aspects are considered, Arrhenius type analyses are typically applied phenomenologically to emission intensities or decay rates associated with thermal quenching processes, providing limited insight into the elementary steps controlling aggregate stability.^16,17^ Consequently, it remains unclear whether AIE aggregation in gold­(I) systems proceeds through discrete nucleation and growth pathways, multistep hierarchical assembly, or continuous reorganization processes governed by a single dominant energetic barrier. This limitation is particularly acute in microheterogeneous solvent environments, where local solvent composition and dynamics can modulate encounter probabilities, interer unit interactions and self-assembly pathways. The absence of quantitative time and temperature resolved kinetic studies capable of discriminating between discrete multistep aggregation and continuous distributed reorganization processes has hampered the rational design of thermoresponsive Au­(I) based AIE materials with controlled stability, reversibility, and long-term photostability. Addressing this gap is essential for advancing the predictive understanding of metallophilic self-assembly and its impact on luminescence performance and requires the application of suitable chemometric tools capable of extracting chemically meaningful and kinetically consistent profiles from highly overlapping spectroscopic data.

Herein, we present a comprehensive kinetic investigation of an AIE-active heterometallic Au­(I)/Ag­(I) tetranuclear complex in acetonitrile/water mixtures. By combining time- and temperature-resolved absorption spectroscopy, emission monitoring, precipitation analysis and multivariate curve resolution (MCR) analysis to extract consistent concentration profiles, we quantitatively resolve the temporal evolution of the aggregation process across a given temperature range. The extracted rate constants exhibit a well-defined Arrhenius behavior governed by a single effective activation energy, which closely agrees DFT BSSE-corrected interaction energies between individual tetranuclear units. This finding strongly supports the assignment of interunit aurophilic and aromatic interactions as the rate-determining step of aggregation. Moreover, the absence of spectrally distinguishable intermediates, as evidenced by the multivariate analysis of the time-resolved data, is consistent with a continuous reorganization pathway rather than a classical nucleation-driven mechanism. These results establish a quantitative kinetic and energetic framework for aggregation-induced emission in gold-based systems operating in microheterogeneous solvent environments, providing fundamental insight into aggregate stability and guiding the rational design of next-generation thermoresponsive metallophilic luminogens.

## Results

### Aggregation-Induced Photophysical Properties

The heterometallic complex [Au_2_Ag_2_(C_6_F_5_)_4_(N≡CCH_3_)_2_]_n_ was previously shown to display bright solid-state luminescence arising from its tetranuclear [Au_2_Ag_2_] cores interconnected through aurophilic interactions.^18^ In the solid state at room temperature, the compound exhibited an intense yellow emission centered at ca. 556 nm upon excitation with UV light, which appeared red-shifted to approximately 567 nm at 77 K, indicative of a rigid, crystallization-stabilized emissive state. Lifetime measurements in the nanosecond regime pointed to spin-allowed transitions. While the complex was essentially nonemissive in dilute acetonitrile solutions at room temperature, increasing concentration led to pronounced deviations from Beer–Lambert behavior and the emergence of low-energy absorption features, consistent with aggregation and oligomer formation mediated by Au···Au interactions. This aggregation process resulted in excitation and absorption spectra that progressively resembled that of the solid state crystalline arrangement.^19^ Solid-state diffuse reflectance measurements further revealed a distinct low-energy absorption band (ca. 490 nm) absent in mononuclear precursors, which was assigned to electronic transitions involving orbitals localized on the interacting tetranuclear metal cores. Time-dependent DFT calculations supported this interpretation, showing that the emissive transitions originated from HOMO–LUMO excitations with substantial Au–Au and Au–Ag metal–metal interaction character, thereby establishing a direct structure–photophysics relationship in this type of systems.

Building on the previously observed concentration-dependent oligomerization of this system in acetonitrile, the aggregation-induced emission (AIE) behavior of [Au_2_Ag_2_(C_6_F_5_)_4_(N≡CCH_3_)_2_]_n_ was next examined using acetonitrile:water mixtures (1 × 10^–4^ M) as a controlled means to promote aggregation. Progressive addition of water to the acetonitrile solution of the complex leads to marked changes in the absorption spectrum. Thus, whereas solutions with higher acetonitrile content display predominantly high-energy ligand-centered bands, mixtures containing 75–80% water give rise to an intense low-energy absorption centered at ca. 485 nm, closely resembling the band observed previously in the diffuse-reflectance spectrum of the crystalline solid. Concomitantly, the emission response evolves from virtually silent to strongly emissive, with a pronounced green luminescence centered at ca. 540 nm emerging for water-rich mixtures with water content above 75%, thus evidencing a clear AIE response (Figure 1).

**Figure 1:**
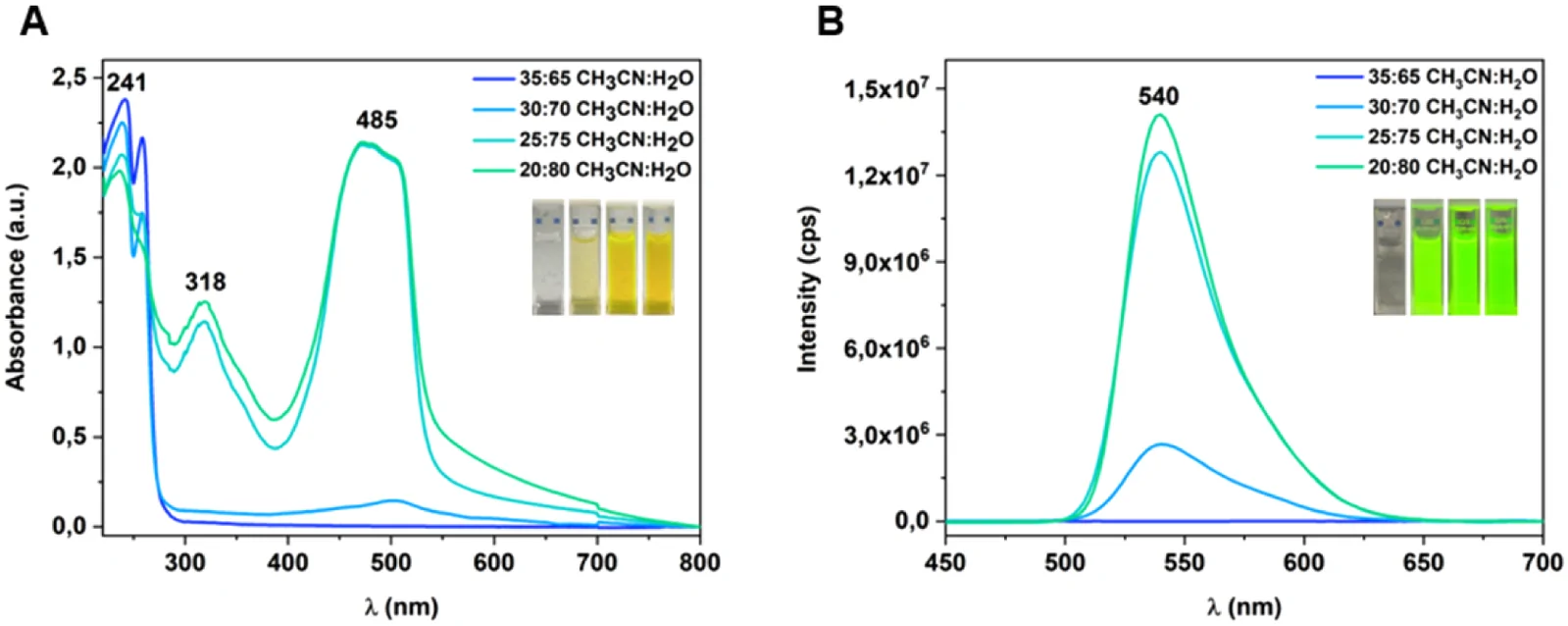
(A) UV–vis absorption spectra of [Au_2_Ag_2_(C_6_F_5_)_4_(N≡CCH_3_)_2_]_n_ recorded in acetonitrile/water mixtures with increasing water content (from 35:65 to 20:80, v/v), showing the emergence of a pronounced low-energy absorption band around 485 nm at high water fractions, characteristic of aggregated species. (B) Corresponding emission spectra of the same solutions, highlighting a strong AIE centered at ca. 540 nm for water-rich mixtures, whereas acetonitrile-rich solutions display weak or negligible luminescence.

Concentration-dependent studies performed at a fixed 20:80 acetonitrile:water ratio further confirm the aggregation-driven nature of this behavior. Highly dilute solutions (e.g., 2 × 10^–5^ M) are poorly emissive, while increasing the concentration results in a progressive enhancement of both the low-energy absorption at 485 nm and the emission intensity of the band at 540 nm, even at moderate concentrations on the order of 10^–4^ M (see Figure 2). The parallel evolution of absorption and emission features with solvent composition and concentration rules out simple solvatochromic effects and directly connects these observations with the aggregation processes previously identified in pure acetonitrile.^18^ The lifetime of the emissive AIE species is 374 ± 9 ns, displaying a similar order of magnitude to the previously measured for complex [Au_2_Ag_2_(C_6_F_5_)_4_(N≡CCH_3_)_2_]_n_ in solid state, suggesting a fluorescent behavior. In addition, this highly emissive AIE state shows a high quantum yield of 55%.

**Figure 2:**
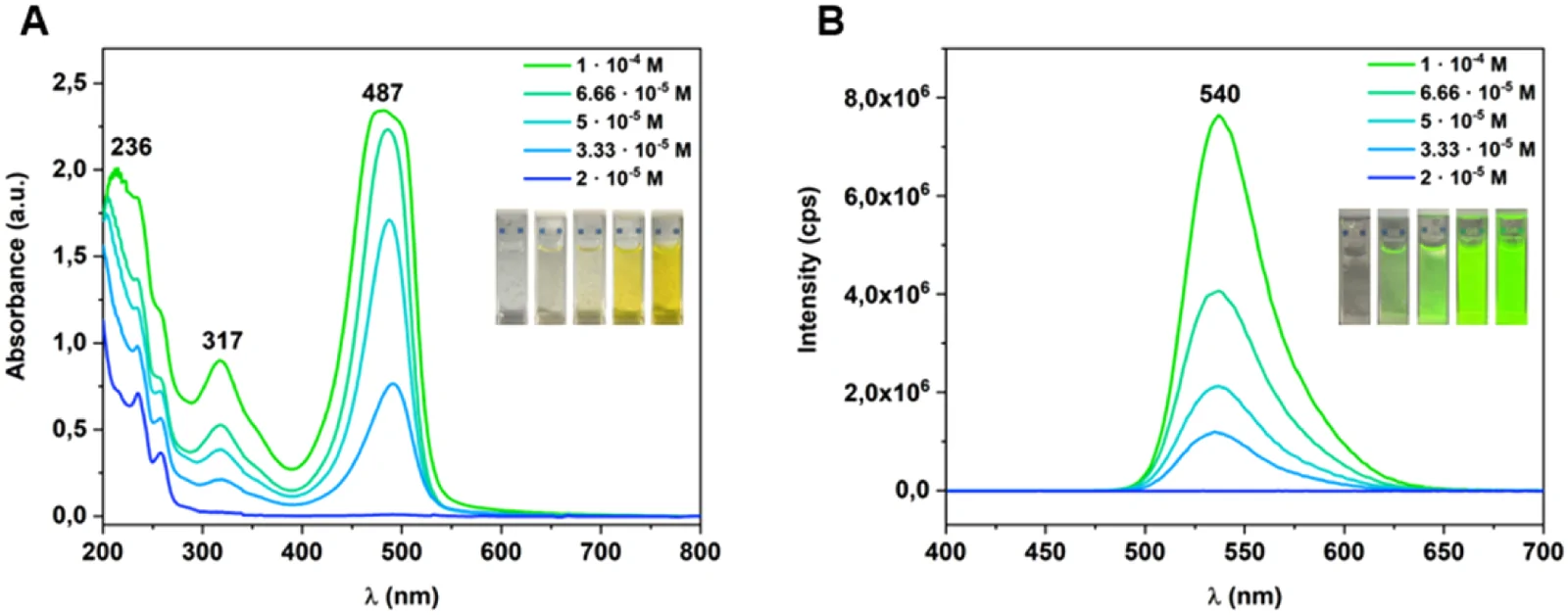
(A) UV–vis absorption spectra of [Au_2_Ag_2_(C_6_F_5_)_4_(N≡CCH_3_)_2_]_n_ recorded at different concentrations (from 2 × 10^–5^ to 1 × 10^–4^ M) in a fixed acetonitrile/water mixture (20:80, v/v), showing the progressive growth of a low-energy absorption band centered at ca. 487 nm with increasing concentration, indicative of aggregate formation. (B) corresponding emission spectra, highlighting a marked enhancement of the aggregation-induced emission centered at ca. 540 nm as the concentration increases, while dilute solutions remain weakly emissive or nonemissive.

### Temperature-Dependent Evolution and Kinetic Stability of Aurophilic AIE Aggregates

To gain insight into the dynamic thermal stability and assembly/disassembly pathways of the aurophilic AIE aggregates, we monitored the temporal evolution of the UV–vis absorption spectra of [Au_2_Ag_2_(C_6_F_5_)_4_(N≡CCH_3_)_2_]_n_ in acetonitrile/water (20:80 v/v) at a 1 × 10^–4^ M concentration and at five closely spaced temperatures between 22 and 28 °C. This solvent composition was selected because, as we have observed, it maximizes the responses of intensity of absorption and emission attributed to the aggregation-induced process, while maintaining sufficient optical transparency to follow aggregate evolution spectroscopically (see Supporting Information).

Immediately after sample preparation (t = 0 min), all experiments display the characteristic low-energy absorption band centered at ca. 485–500 nm, previously identified as the spectroscopic signature of aggregated tetranuclear species linked by Au···Au interactions and closely resembling the diffuse reflectance band observed in the crystalline solid state.

Upon standing at constant temperature, a gradual but systematic decrease in the intensity of this low-energy band with time is observed. Importantly, no concomitant increases of sharp high-energy ligand-centered bands are detected, ruling out simple molecular dissolution into monomeric species. Instead, the uniform attenuation of absorbance is consistent with a progressive precipitation taking the aggregates out of the optical path. This interpretation is visually corroborated by the time-resolved photographs of the solutions at 25 °C, which reveal gradual clarification of the supernatant and the formation of sediment over time, in agreement with the spectroscopic trends (Figure 3).

**Figure 3:**
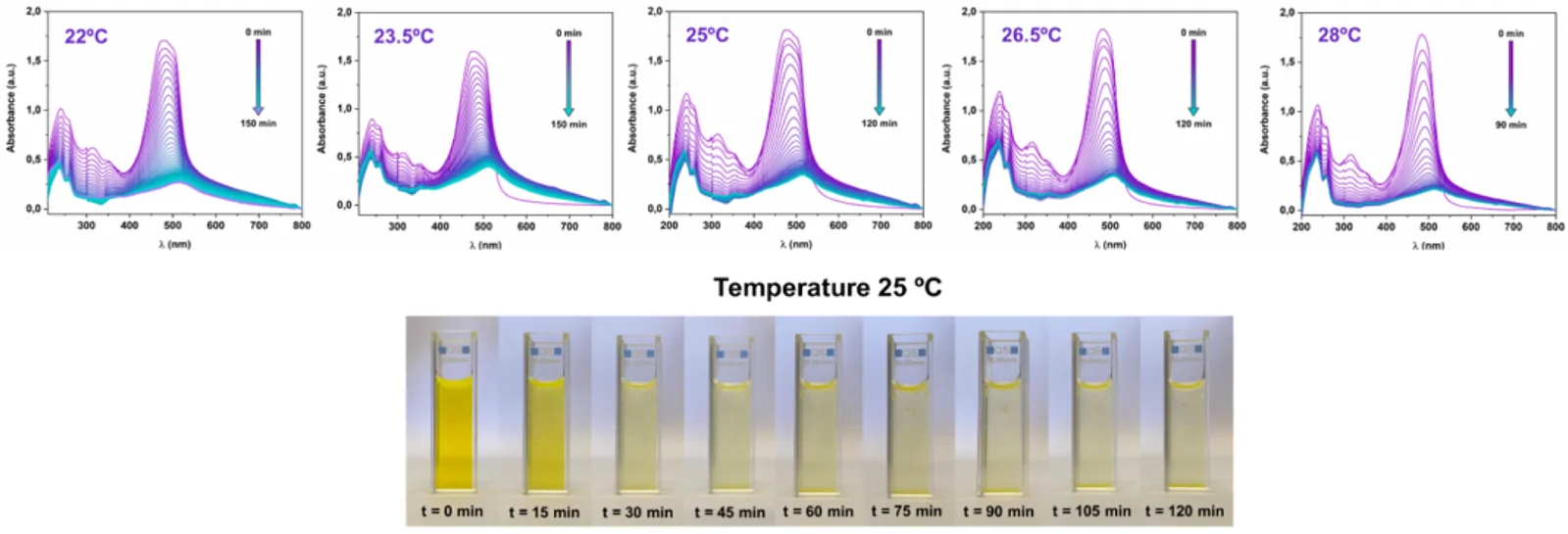
Time-resolved UV–vis absorption spectra of [Au_2_Ag_2_(C_6_F_5_)_4_(N≡CCH_3_)_2_]_n_ recorded in acetonitrile/water (20:80, v/v) at different temperatures (22, 23.5, 25, 26.5, and 28 °C), showing the progressive attenuation of the low-energy absorption band. The temporal evolution (from *t* = 0 to 90–150 min, depending on temperature) highlights an accelerated decay of the aggregated species with increasing temperature. Bottom panel: photographic images of the solution at 25 °C at selected times, illustrating the gradual discoloration of the solution and the formation of precipitate.

Notably, the rate of decay of the low-energy absorption band is strongly temperature dependent. At 22 °C, the aggregation induced emissive state exhibits comparatively higher kinetic stability, with the characteristic absorption persisting for up to 150 min before significant attenuation occurs. In contrast, increasing the temperature by only a few degrees markedly accelerates the process: at 25–26.5 °C, comparable spectral attenuation is observed within ca. 120 min, while at 28 °C the low-energy band collapses within roughly 90 min. The emission spectra appear partially attenuated following a similar trend (see Supporting Information for the time-dependent emission at 25 °C).

The temporal evolution of aggregate size and the concentration-dependent aggregate growth was investigated by dynamic light scattering (DLS) for [Au_2_Ag_2_(C_6_F_5_)_4_(N≡CCH_3_)_2_]_n_ acetonitrile/water (20:80 v/v) at concentrations of 1 × 10^–4^ M and 3.3 × 10^–5^ M, under conditions identical to those employed for the UV–vis kinetic experiments (see Supporting Information). At 1 × 10^–4^ M, DLS measurements performed immediately after sample preparation (t = 0 min) reveal the presence of relatively small aggregates with a characteristic hydrodynamic diameter of ca. 60 nm, which dominate the number-weighted distribution. These early-stage species are consistent with primary AIE-active aggregates inferred from the appearance of the low-energy absorption band and strong emission. Even at these short times, however, the intensity-weighted distributions already show contributions from larger objects displaying much higher light dispersion ability approaching 1 μm, indicating the rapid onset of aggregate-aggregate interactions.

As the system evolves, a clear and systematic growth process is observed. After 10–15 min, the dominant population shifts toward submicron to micron-sized aggregates (ca. 0.8–1.2 μm), with larger species (ca. 3.3–9.3 μm) contributing disproportionately to the scattering intensity while remaining a minor fraction in number. By 20 min, both the intensity-weighted and number-weighted distributions are largely governed by aggregates with hydrodynamic diameters of ca. 3 μm. At longer times (30–35 min), very large aggregates with characteristic dimensions of several micrometers (up to ca. 5–10 μm) dominate the intensity and volume distributions, concomitant with a substantial depletion of smaller species and signaling the onset of sedimentation and loss of optically active material from the scattering volume (Scheme 1). The spectroscopic analysis of the obtained final aggregate species agrees with the initial product, as reflected in the corresponding FTIR and ^19^F­{^1^H} NMR spectra (see Supporting Information).

**Scheme 1:**
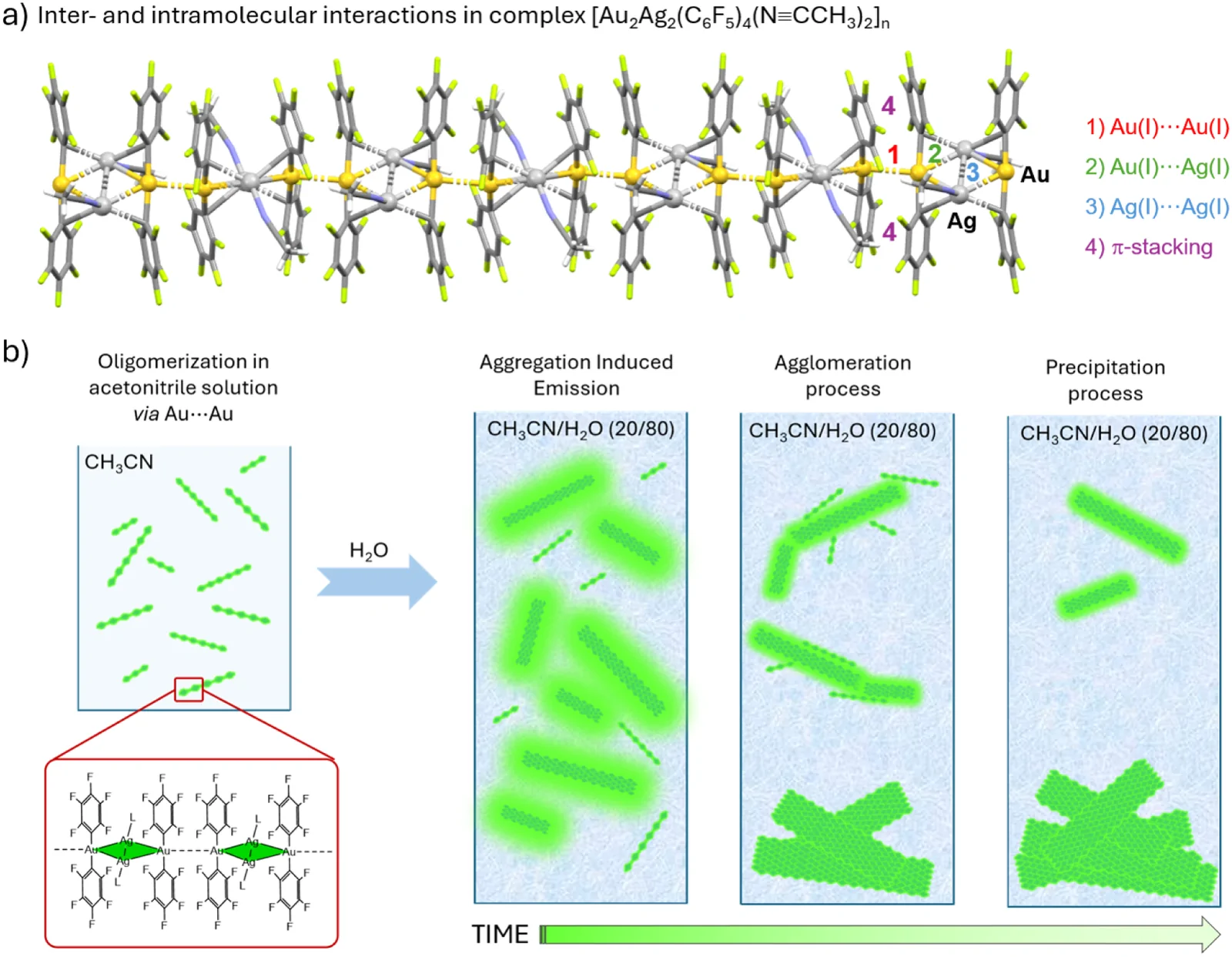
a) Representation of the X-ray Diffraction 1D Structure of Complex [Au_2_Ag_2_(C_6_F_5_)_4_(N≡CCH_3_)_2_]_n_ Displaying the Most Important Inter- and Intramolecular Interactions (See Ref 15). b) (Left) Oligomerization Process in Acetonitrile Solution with Increasing Concentrations of Complex [Au_2_Ag_2_(C_6_F_5_)_4_(N≡CCH_3_)_2_]_n_ (Right) Schematic Description of the AIE Process in CH_3_CN/H_2_O (20:80 v/v) and Subsequent Agglomeration and Precipitation Processes Showing Decreasing Absorption/Emission Intensity with Time

In sharp contrast, the DLS data collected for the dilute solution (3.3 × 10^–5^ M) show markedly different behavior. At early times, weak scattering features corresponding to small aggregates or loose clusters in the 300–800 nm range are occasionally detected, but these species are short-lived. Over the entire time window investigated, no sustained growth toward micron-sized aggregates is observed. Instead, the distributions rapidly converge toward either very small sizes near the detection limit or highly transient clusters that do not evolve into stable larger assemblies. Even at longer times (up to 12 min), intensity-weighted distributions fail to show the pronounced shift toward micrometer-scale objects that characterizes the 1 × 10^–4^ M solution, indicating that aggregate-aggregate fusion is strongly suppressed at low concentration. Importantly, in this case precipitation is not observed.

To gain kinetic mechanistic insight, the time- and temperature-dependent evolution of the aggregated absorbing species was analyzed by applying a multivariate curve resolution-alternating least-squares (MCR-ALS) methodology in combination with kinetic fitting.^20–22^ Due to the strong spectral overlap across the full temporal window, direct single-wavelength analysis was not reliable, and concentration profiles were therefore extracted through multivariate resolution, enabling explicit separation of overlapping spectral contributions. Prior to multivariate analysis, the UV–vis spectra were pretreated using asymmetric least-squares (AsLS) baseline correction and multiplicative scatter correction (MSC) to minimize additive and multiplicative background contributions, ensuring that the remaining variations mainly reflect underlying chemical changes. Global analysis of the concatenated time-resolved UV–vis data sets recorded at five temperatures (22.0, 23.5, 25.0, 26.5, and 28.0 °C) yielded a stable decomposition into three components, two of which were assigned to chemically meaningful species corresponding to the initial confined state and the reorganized aggregate, while the third accounts for residual spectral variance and was required to ensure numerical stability of the solution (Figure 4).

**Figure 4:**
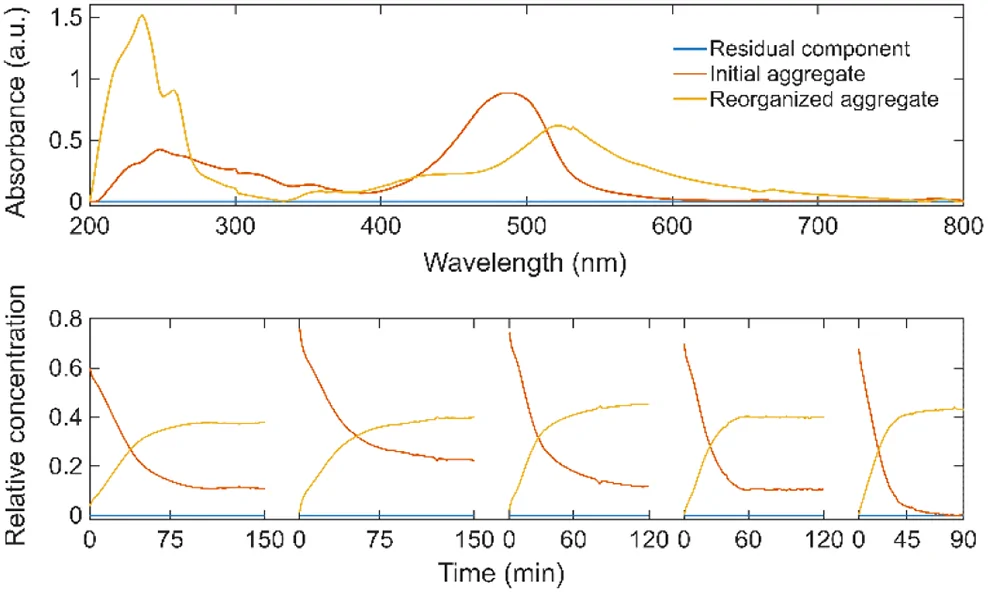
(top) Spectral profiles and (bottom) corresponding concentration profiles obtained by MCR-ALS for the time-resolved data series.

The robustness of this solution was further validated through a combination of complementary analyses, including exploration of higher-order decompositions and cross-comparison of resolved spectral profiles across temperature blocks, none of which revealed additional chemically meaningful components. The MCR-ALS analysis was performed under constrained conditions, including non-negativity on both concentration and spectral profiles, together with selectivity constraints defined in specific spectral regions where one component is known to dominate, based on the observed spectral evolution. Constraints were introduced in the low-wavelength region primarily associated with the initial state and in the red-shifted region characteristic of the reorganized aggregate. In addition, mild spectral weighting was applied to reduce the influence of regions affected by lower signal-to-noise ratios, and regularization was used to further stabilize the solution and prevent overfitting. The concentration profile of the reorganized aggregate shows smooth, monotonic growth as a function of time at all temperatures, with no evidence for the appearance of additional spectral species. It is worth noting that no kinetic constraints were imposed during the resolution step, allowing the concentration profiles to be extracted solely from the spectral information without incorporating mechanistic assumptions.

Importantly, the MCR-ALS results exclude the presence of discrete reaction intermediates: no isosbestic points, induction periods, abrupt kinetic regime changes, or coexistence of multiple well-defined rate constants are detected. Consistently, the extracted concentration profiles are smooth and unimodal, without inflection points or features indicative of sequential regimes. These features could rule out a classical nucleation–growth mechanism or a sequential multistep aggregation pathway involving structurally distinct intermediates. Instead, the experimental data are fully consistent with a continuous reorganization process governed by a distribution of closely related kinetic barriers.

To account for this behavior, the temporal evolution of the concentration of the reorganized aggregates, as obtained from the MCR-ALS analysis, was analyzed by systematically comparing alternative kinetic models (see Supporting Information), from which a distributed kinetic expression, specifically a Kohlrausch–Williams–Watts (KWW) or stretched-exponential function,^23,24^

$$C\left(t\right)=C_{0}+C_{\infty }\left[1-e^{-{\left(kt\right)}^{\beta }}\right]$$

where k is an effective kinetic constant and β is the stretching exponent, emerges as the most suitable model to account for the observed kinetic behavior. In the present system, the fitted values of β are consistently slightly larger than unity for all temperatures investigated. This places the system in the regime of compressed-exponential kinetics, rather than the more common stretched regime (β < 1) associated with static disorder or broad distributions of independent relaxation events (Figure 5).

**Figure 5:**
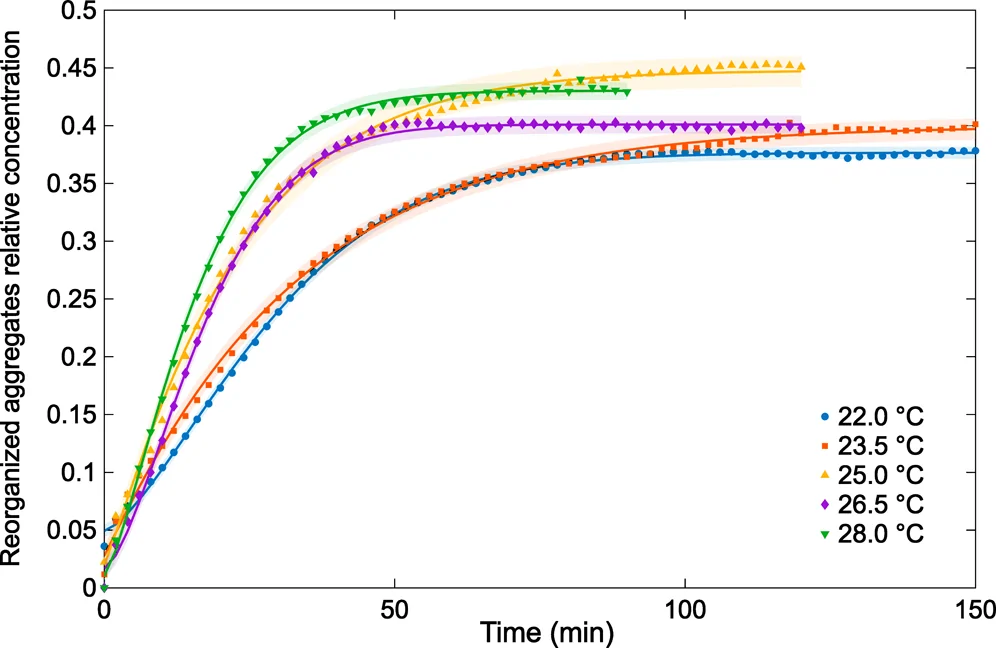
Concentration profiles of the reorganized aggregates and corresponding fits obtained using the offset-corrected KWW (compressed-exponential) model, identified as the optimal description across all temperatures. Symbols represent the MCR-ALS-resolved concentration profiles, solid lines the fitted profiles, and shaded regions the 95% confidence intervals.

Values of β > 1 are characteristic of cooperative and correlated relaxation processes, in which configurational reorganization proceeds faster than expected for ideal first-order kinetics. In the context of the present system, this behavior indicates that aggregate reorganization is not governed by independent decay of isolated entities, but instead by collective aggregate-aggregate interactions. Structural rearrangement within one aggregate promotes or accelerates the evolution of neighboring aggregates, giving rise to correlated dynamics across the ensemble.

The observation of compressed-exponential kinetics, together with the absence of discrete spectral intermediates, rules out nucleation-limited aggregation or sequential bond-breaking/formation mechanisms. Instead, it supports a scenario in which AIE-active aggregates undergo a cooperative and continuous structural evolution, driven by gradual weakening, reorganization, and redistribution of Au···Au metallophilic interactions that leads to the formation of large aggregates that precipitate. Within this framework, the effective rate constant k represents an Arrhenius-averaged measure of the collective reorganization rate, while the value of β > 1 reflects internal mechanical or packing constraints within the aggregates that transiently store configurational stress and release it through concerted restructuring and growth.

Notably, the stretching exponent β remains essentially invariant with temperature, indicating that thermal activation accelerates the kinetics without altering the underlying mechanistic nature of the process. This invariance confirms that the same cooperative reorganization pathway operates across the entire temperature range studied.

The temperature dependence of the extracted rate constants was analyzed using an Arrhenius formalism, yielding a linear Arrhenius plot and suggesting that the process is thermally activated and kinetically controlled (see Figure 6). From this analysis, an activation energy of *E*_a_ = 86.0 kJ·mol^–1^ was obtained, with a very narrow confidence interval, underscoring the robustness of the kinetic model and the internal consistency of the fitting. The magnitude of this activation energy is consistent with a process involving partial disruption and reorganization of cooperative metallophilic networks, rather than simple diffusive dissolution or monomer release.

**Figure 6:**
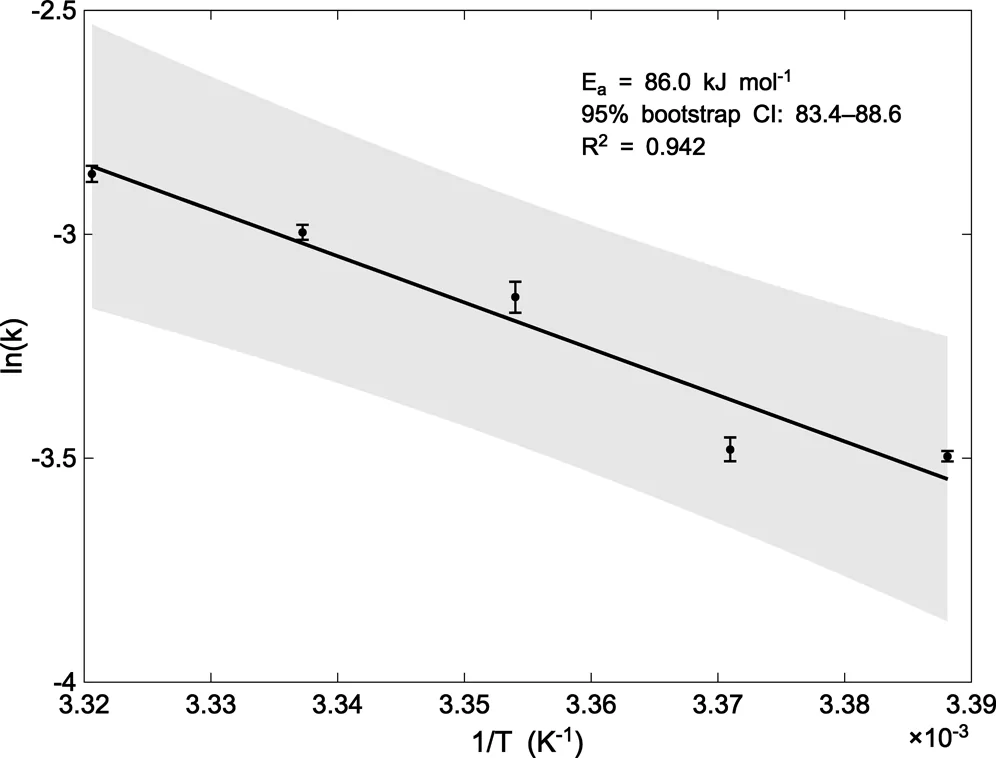
Arrhenius plot of the effective rate constants obtained from the offset-corrected KWW model. Error bars correspond to 95% bootstrap confidence intervals obtained from 2000 resamples of the kinetic fits. The solid line represents the linear Arrhenius regression, while the shaded region denotes the 95% prediction band. The fit yields an extracted activation energy of 86.0 kJ mol^–1^ (95% bootstrap CI: 83.4–88.6 kJ mol^–1^; R^2^ = 0.942).

The Arrhenius representation exhibited good linearity over the investigated temperature interval (R^2^ = 0.942). To quantify parameter uncertainty, bootstrap resampling (2000 replicates per temperature) was applied to the fitted KWW-offset kinetic profiles. Thus, multiple synthetic data sets were generated by randomly resampling the residuals of the original kinetic fits and adding them back to the fitted profiles, thereby preserving the level of experimental noise observed in the measurements. Refitting these synthetic data sets yielded distributions of the kinetic constants and, subsequently, of the Arrhenius parameters. This procedure resulted in an effective activation energy of 86.0 kJ mol^–1^ with a 95% confidence interval of 83.4–88.6 kJ mol^–1^. The close agreement between the nominal and bootstrap-derived activation energies, together with the narrow confidence interval obtained, supports the robustness of the extracted activation parameter within the investigated temperature range.

Although the investigated temperature window is relatively narrow (295–301 K), no evidence of departures from Arrhenius behavior was observed within this interval. Consequently, the reported activation energy should be regarded as an effective activation parameter describing the temperature domain explored in this work.

To further rationalize the origin of the kinetic barrier associated with the reorganization process, density functional theory (DFT) calculations were performed on a dimeric model system, [Au_2_Ag_2_(C_6_F_5_)_4_(N≡CCH_3_)_2_]···[Au_2_Ag_2_(C_6_F_5_)_4_(N≡CCH_3_)_2_], in which two tetranuclear units are held together through a combination of a single aurophilic Au···Au interaction and intermolecular π–π contacts between perfluorophenyl ligands. The interaction energy estimated using the counterpoise correction to account for basis set superposition error (BSSE),^25^ was found to be 116 kJ·mol^–1^. This value and the determined activation energy for the thermally induced reorganization process (*E*_a_ = 86 kJ·mol^–1^) are of the same order of magnitude.

In addition, Conductor-like Screening Model (COSMO) calculations^26^ performed at DFT level in water provide a solvent-dependent representation of the electrostatic environment at the solute ([Au_2_Ag_2_(C_6_F_5_)_4_(N≡CCH_3_)_2_]_2_ model) -solvent (water) interface (Figure 7).

**Figure 7:**
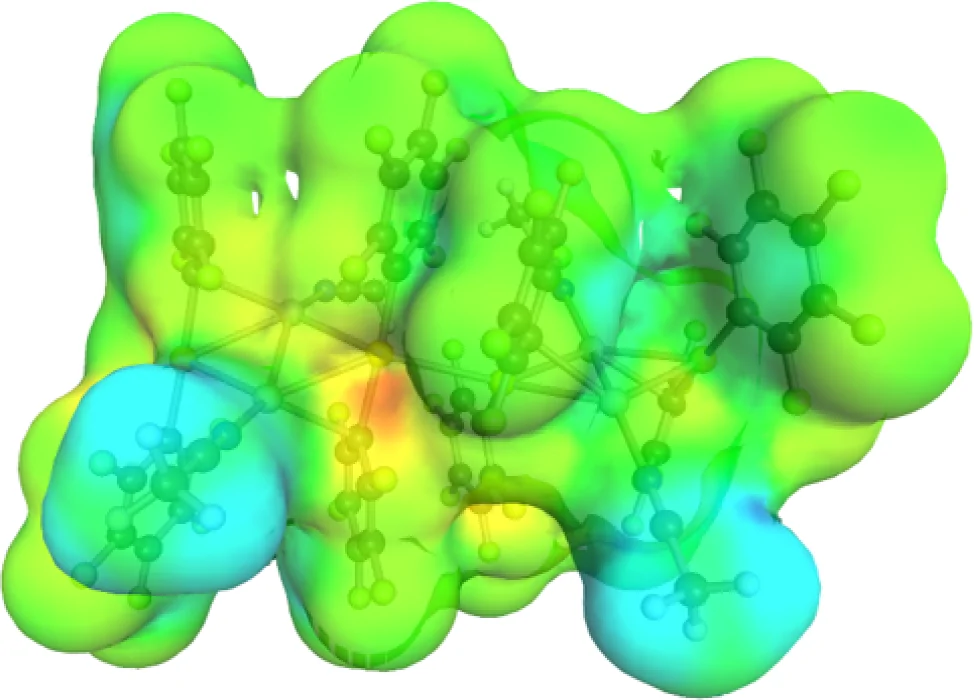
Conductor-like screening model (COSMO) isosurface (DFT density) of [Au_2_Ag_2_(C_6_F_5_)_4_(N≡CCH_3_)_2_]_2_ model in water displaying a solvent-dependent representation of the electrostatic environment at the solute–solvent interface.

COSMO calculations provide a solvent-dependent representation of the molecular surface in terms of its screening charge density, reflecting the polarization of the surrounding medium and thereby the ability of different regions of the molecule to interact with the solvent. In this framework, strongly colored regions (blue for excess of electron density and red for defect of electron density) correspond to sites capable of engaging in favorable solute–solvent interactions, whereas green areas represent nearly neutral surface regions with limited interaction capability. In the present case, the COSMO surface in water is predominantly characterized by such neutral (green) regions, indicating a globally weak and inefficient solvation of the molecular periphery. This limited solvent stabilization implies that the energetic gain associated with solute–solvent interactions is relatively small, allowing intermolecular interactions, such as aurophilic Au···Au contacts and π-π stacking between C_6_F_5_ rings, to become competitive or even dominant. Consequently, the balance between solvation and self-association is shifted toward intermolecular aggregation, providing a molecular-level rationale for the experimentally observed assembly, cooperative growth, and eventual precipitation of the AIE-active aggregates. The dominance of intermolecular interactions inferred from the COSMO surface agrees with the DFT computed interaction energy between tetranuclear units, also comparable to the experimental activation barrier, thereby confirming the central role of aurophilic interactions in the aggregation and precipitation process.

## Discussion

The spectroscopic results provide direct and consistent evidence that the emissive state of [Au_2_Ag_2_(C_6_F_5_)_4_(N≡CCH_3_)_2_]_n_ is associated with the formation of aggregated species stabilized by metallophilic interactions and π-π stacking. The appearance of a well-defined low-energy absorption band at ca. 485–500 nm, together with the emergence of intense emission centered at ca. 540 nm, constitutes a clear spectroscopic demonstration of the existence of these aggregates. Importantly, both features evolve in parallel with solvent composition (Figure 1) and concentration (Figure 2), confirming that the optical response is governed by aggregation rather than solvatochromic effects.

The fact that the position and shape of the low-energy band closely match those observed in the diffuse reflectance spectrum of the solid indicates that the electronic structure of the aggregated species in solution already approaches that of the crystalline phase. This observation provides strong support for a model in which aggregation induces the formation of electronically coupled tetranuclear units, where Au···Au and Au···Ag interactions give rise to new metal–metal–ligand excited states, consistent with the TD-DFT calculations.^18,19^

Another essential fact is the strong dependence of aggregation on solvent composition, which highlights the critical role of the acetonitrile/water medium. At high water fractions (≥75%), the system operates within a microheterogeneous regime, where nanoscale fluctuations in solvent composition create transient domains of differing polarity.

Previous structural and spectroscopic studies of acetonitrile/H_2_O mixtures in similar composition ranges have shown that these systems exhibit pronounced nanoscale compositional fluctuations, with characteristic domain sizes typically in the subnanometer to nanometer regime, arising from the segregation of hydrogen-bonded water clusters and dipolar acetonitrile-enriched regions. Within this context, the rapid appearance at t = 0 min of aggregated species, together with the detection by DLS of primary particles with hydrodynamic diameters of ca. 60 nm, suggests that these initial aggregates are not formed in a homogeneous medium, but rather emerge from the coalescence of multiple solvent nanodomains acting as transient confinement environments. The size mismatch between the intrinsic solvent domains (subnanometric) and the experimentally observed nanoaggregates (tens of nanometers) therefore reflects a hierarchical organization process, in which localized molecular enrichment within acetonitrile-rich regions promotes nucleation, while subsequent domain fusion and interdomain exchange enable the growth of stable nanoscopic aggregates. This interpretation is consistent with the absence of induction periods in the kinetic traces and with the immediate development of the AIE-active spectral signature, both of which indicate that aggregation is seeded by preexisting solvent structure rather than by classical homogeneous nucleation in bulk solution.

Within this environment, the formation of aggregates can be rationalized as a domain-assisted process. Acetonitrile-rich regions act as preferential solvation environments that locally concentrate the complex, thereby facilitating interunit encounters and the formation of initial nanoaggregates. At the same time, the surrounding hydrogen-bonded aqueous network imposes a solvophobic driving force that promotes further association by expulsion from the bulk phase. This interpretation is consistent with the immediate appearance, at t = 0 min, of the low-energy absorption band characteristic of aggregated species (Figure 3), indicating that aggregation is not kinetically delayed, but instead occurs rapidly upon mixing. The acetonitrile domains solvent, therefore, does not merely induce aggregation, but actively define the spatial and energetic landscape in which self-assembly takes place.

With these assumptions, spectroscopic time-resolved evolution suggests a process of continuous growth and precipitation. Thus, the time-resolved UV–vis experiments provide a detailed picture of the evolution of the aggregated state. At all investigated temperatures (22.0–28.0 °C), the system initially displays a strong low-energy absorption band, which subsequently decreases smoothly with time over a characteristic time scale that depends on temperature (from ca. 150 min at 22 °C to ca. 90 min at 28 °C). The main features are (i) the decay is continuous and monotonic at all temperatures; (ii) no new bands appear; (iii) no recovery of high-energy monomer-like features is observed; (iv) and no isosbestic points are detected.

These observations could rule out a simple equilibrium between aggregated and monomer/oligomer molecular species, while instead point to a progressive grow and subsequent loss of aggregates from the solution phase via precipitation. This interpretation is fully supported by DLS data. At 1 × 10^–4^ M concentration, at early times the system initially consists of nanoaggregates with hydrodynamic diameters of ca. 60 nm (number-weighted distribution). However, the intensity-weighted distributions reveal the presence of larger species approaching ca. 1 μm, indicating the onset of aggregate-aggregate interactions. As time evolves (at 10–15 min), we observe dominant populations shift to 0.8–1.2 μm; at 20 min, aggregates of ca. 3 μm; and at 30–35 min, very large objects (5–10 μm) are observed. This continuous growth directly correlates with the attenuation of the low-energy absorption band, suggesting that the spectroscopic signal is lost not because of electronic quenching, but because aggregates grow beyond the colloidal stability limit and sediment out of the optical path. In addition, within the same experiment, the comparison between concentrations reveals a clear kinetic threshold. While the 1 × 10^–4^ M solution undergoes rapid aggregate growth leading to precipitation, the more dilute system (3.3 × 10^–5^ M) exhibits a different behavior. Thus, aggregates remain in the submicron or nanometric range; no sustained growth toward micrometer sizes is observed; and precipitation does not occur. This different behavior supports that aggregation proceeds via aggregate-aggregate fusion processes that are strongly concentration-dependent, rather than solely by monomer addition. Below a critical concentration, these interactions are inefficient to extend in larger aggregates, and the system remains trapped in a regime of small, transient clusters in the acetonitrile/water domains.

One of the most significant aspects of the present study is the remarkable consistency of the kinetic behavior across all temperatures investigated. The comprehensive chemometric approach applied here reveals that the same spectrally resolved components and associated transformation pathway are consistently recovered across all temperatures, indicating that the underlying process remains invariant under all conditions studied. In fact, the kinetic traces at all temperatures can be described using a single functional form, namely compressed-exponential time dependence (KWW model),^23,24^ without the need to introduce additional phenomenological terms or multistep mechanisms. This kinetic description is not imposed a priori, but results from a broad, nonbiased multimodel fitting procedure, in which a range of kinetic models (from simple exponential to nucleation- and growth-type models) were evaluated against the concentration profiles obtained from the MCR-ALS analysis (see Supporting Information).^20–22^ In combination with the absence of kinetic (hard) constraints during the resolution step, this ensures that both the concentration profiles and their temporal evolution are derived exclusively from the spectral data, without embedding predefined mechanistic assumptions. The fits are consistently robust and internally coherent across the full data set, supporting that the proposed model captures the key features of the system evolution. The absence of kinetic induction periods, multirate regimes, or deviations from the model behavior strongly supports a compact and robust mechanistic description. An important fact is that the fitted values of the stretching exponent β are consistently greater than unity (β > 1) for all temperatures. This observation is highly diagnostic. While β < 1 is typically associated with static disorder and independent relaxation processes, β > 1 indicates cooperative and correlated dynamics, in which the evolution of individual aggregates is strongly coupled to that of their surroundings.

In the present system, this behavior is fully consistent with the DLS results, which show that aggregation proceeds through aggregate-aggregate interactions, rather than independent decay events. Structural rearrangements within one aggregate facilitate and promote the evolution of neighboring aggregates, resulting in a collective reorganization process that proceeds faster than expected for simple first-order kinetics.

Importantly, the value of β remains invariant across the entire temperature range, indicating that temperature accelerates the kinetics without altering the underlying mechanism. This invariance is a key result, as it suggests that the same cooperative reorganization pathway operates from 22 to 28 °C.

The temperature dependence of the effective rate constants exhibits a strictly linear Arrhenius behavior, yielding an activation energy of *E*_a_ = 86.0 kJ·mol^–1^ with minimal dispersion of the experimental data, confirming the robustness of the kinetic model. To gain insight into the origin of this barrier, the interaction energy between two tetranuclear units was estimated by DFT calculations, giving a value of approximately 116 kJ·mol^–1^. While activation energies and interaction energies describe fundamentally different physical quantities, their similar magnitude is highly informative. Importantly, the aggregation process observed here does not require complete dissociation of preformed units followed by reassociation but is better described as a continuous and cooperative reorganization occurring within a microheterogeneous solvent environment. In this scenario, aggregate growth proceeds through acetonitrile domain-mediated encounters and structural rearrangements, in which Au···Au and π-π interactions are dynamically redistributed rather than fully broken. Consequently, the activation energy should be interpreted as an effective barrier associated with collective reorganization under confinement, encompassing solvent reconfiguration, domain coalescence, and progressive adjustment of metallophilic contacts. The agreement in order of magnitude between the experimental *E*_a_ and the computed interaction energy therefore indicates that the dynamics are governed by the same energetic scale defined by interunit metallophilic coupling, without implying a direct equivalence or a discrete bond-breaking mechanism. This interpretation is further supported by the absence of identifiable intermediates and by the compressed-exponential kinetics, both of which point to a cooperative and spatially correlated evolution of the aggregated state.

COSMO surface calculations agree with a weak solvation of the molecular periphery, precluding solute–solvent stabilizing interactions, what allow intermolecular aurophilic and π-π stacking interactions.

Combining all experimental and theoretical evidence, a coherent mechanism can be deduced. First, a rapid initial aggregation in microheterogeneous domains, yielding AIE-active nanoaggregates (ca. 60 nm). Second, a cooperative growth via aggregate-aggregate interactions, leading to submicron and micrometer structures (0.8–10 μm). This thermally activated reorganization is governed by a single energetic barrier (ca. 86 kJ·mol^–1^). Finally, the progressive sedimentation, causing loss of optical signal. This picture is effective in a range of temperatures (22–28 °C). Within this framework, the AIE state is not a stable end point, but a kinetically persistent intermediate, whose lifetime and observability are controlled by the interplay between metallophilic interactions, solvent organization, and cooperative dynamics. This study can shed light on the internal processes that occur in experiments during the aggregation induced emission processes.

## Conclusions

This study provides a comprehensive kinetic and mechanistic description of aggregation-induced emission (AIE) processes in tetranuclear Au­(I)/Ag­(I) systems in microheterogeneous acetonitrile/water media. By combining time-resolved spectroscopy, chemometric analysis, and DLS measurements, we show that aggregation proceeds via a continuous and cooperative reorganization pathway, rather than a classical nucleation–growth mechanism. The observed compressed-exponential kinetics (β > 1) reveal that correlated aggregate-aggregate interactions dominate the system dynamics, highlighting the collective nature of the reorganization process.

The well-defined activation energy (*E*_a_ = 86 kJ·mol^–1^), obtained from Arrhenius analysis, provides key insight into the energetic landscape governing aggregate evolution. Comparison with DFT calculations shows that interunit interactions (116 kJ·mol^–1^), dominated by aurophilic and π-π stacking forces, define the thermodynamic stabilization of the aggregates and are mechanistically linked to the kinetic barrier, which arises from their reorganization during the aggregation process. This establishes a direct connection between macroscopic kinetics and microscopic interactions, without implying strict equivalence between thermodynamic and activation energies.

In addition, COSMO analysis indicates a globally weak solvation of the molecular interface, rationalizing the reduced contribution of solute–solvent interactions and the resulting dominance of intermolecular forces in driving aggregation. The interplay between weak solvation, metallophilic interactions, and cooperative dynamics ultimately governs the formation, growth, and precipitation of the AIE-active assemblies.

These results provide a unified molecular framework for AIE aggregation dynamics, integrating kinetic, thermodynamic, and solvation effects. Beyond fundamental insight, this work offers design principles for thermoresponsive metallophilic luminogens, where controlled modulation of intermolecular interactions and solvation can be exploited to tune aggregate stability, lifetime, and functional performance.

## Experimental Section

### General Considerations

The starting precursor [Au_2_Ag_2_(C_6_F_5_)_4_(N≡CCH_3_)_2_]_n_ was prepared as described in the literature.^18^ All solvents used for the synthesis of the new compounds were obtained from commercial sources and were employed without further purification.

### Instrumentation

UV–vis spectra of solution samples diluted were recorded on a Shimadzu UV-3600 spectrophotometer equipped with a Peltier device for temperature control. Excitation and emission spectra in solution as well as lifetime measurements were recorded using an Edinburgh FLS 1000 fluorescence spectrometer. DLS measurements were performed on a Brookhaven Instruments Corporation 90 PLUS particle size analyzer on acetonitrile/water mixtures (20:80 ratio) at different concentrations to yield the size distributions by intensity and by number.

### MCR-ALS Analysis

All data analysis was performed using MATLAB (MathWorks, Natick, MA, USA). Multivariate Curve Resolution-Alternating Least Squares (MCR-ALS)^20–22^ was implemented through custom-developed scripts including non-negativity constraints and ridge regularization. Initial spectral estimates were refined using Savitzky-Golay filtering^27^ and local regression smoothing.^28^ Kinetic modeling and uncertainty estimation were carried out using in-house routines based on nonlinear least-squares optimization.

### Computational Details

The optimization of model system [Au_2_Ag_2_(C_6_F_5_)_4_(N≡CCH_3_)_2_]_2_ was performed at the density functional theory (DFT). The BP86 functional^29,30^ was used in combination with the def2-SVP basis set,^31^ and Grimme’s D3­(BJ) dispersion correction^32^ that accounts for van der Waals interactions. The RI approximation^33^ was used to accelerate the evaluation of Coulomb and exact exchange integrals.

The optimization was carried out with the Turbomole 8.0 program package.^34,35^ The Conductor-like Screening Model (COSMO) calculations performed at DFT level in water were carried out using the graphical user interface TmoleX as implemented in Turbomole.^36^

## Supplementary Material



## Notes

Time-dependent emission at 25 °C, DLS and fitted data, solid FTIR, and ^19^F NMR spectrum of the aggregated product (PDF)

## References

[1] Luo J., Xie Z., Lam J. W., Cheng L., Chen H., Qiu C., Kwok H. S., Zhan X., Liu Y., Zhu D. (2001). Aggregation-induced emission of 1-methyl-1,2,3,4,5-pentaphenylsilole. Chem. Commun..

[2] Mei J., Leung N. L. C., Kwok R. T. K., Lam J. W. Y., Tang B. Z. (2015). Aggregation-Induced
Emission: Together We Shine, United We Soar!. Chem. Rev..

[3] Kwok R. T. K., Leung C. W. T., Lam J. W. Y., Tang B. Z. (2015). Biosensing by luminogens
with aggregation-induced emission characteristics. Chem. Soc. Rev.

[4] Alam P., Climent C., Alemany P., Laskar I. R. (2019). “Aggregation-induced
emission” of transition metal compounds: Design, mechanistic
insights, and applications. J. Photochem. Photobiol.,
C.

[5] Feng N., Wang Z., Sun D., Zhang L., Xin X., Sun P., Azam M., Li H. (2024). Kinetically Controlled Structural
Modulation of the Self-Assembled Silver Nanoclusters. Small.

[6] Lia X., Wang W., Li S., Sun M., Li Y., Xu J., Liang X., Sun T., Xin X. (2024). Self-sorting in supramolecular
assembly of water-soluble silver nanocluster/hydrophobic peptides
system. J. Mol. Liq..

[7] Xie H., Wang Y., Zhang N., Li S., Li J., Xin X. (2024). Solvent-Induced
Self-Assembly of Silver Nanoclusters for White-Light-Emitting
Diodes and Temperature Sensing. ACS Appl. Nano
Mater..

[8] Yam V.-W.-W., Au V. K.-M., Leung S. Y.-L. (2015). Light-Emitting Self-Assembled Materials
Based on d8 and d10 Transition Metal Complexes. Chem. Rev..

[9] Pyykkö P. (1997). Strong Closed-Shell
interactions in inorganic chemistry. Chem. Rev.

[10] Pyykkö P. (2004). Theoretical
chemistry of gold. Angew. Chem., Int. Ed..

[11] Pinto A., Svahn N., Lima J. C., Rodríguez L. (2017). Aggregation
induced emission of gold­(i). complexes in water or water mixtures. Dalton Trans..

[12] Cunha C., Pinto A., Galvão A., Rodríguez L., De Melo J. S. S. (2022). Aggregation-Induced Emission with Alkynylcoumarin Dinuclear
Gold­(I) Complexes: Photophysical, Dynamic Light Scattering, and Time-Dependent
Density Functional Theory Studies. Inorg. Chem..

[13] Mountain R. D. (2010). Molecular
Dynamics Study of Water-Acetonitrile Mixtures. J. Phys. Chem. B.

[14] Nagasaka M., Yuzawa H., Kosugi N. (2020). Microheterogeneity in aqueous acetonitrile
solution probed by soft x-ray absorption spectroscopy. J. Phys. Chem. B.

[15] Koley S., Ghosh S. (2015). Study of Microheterogeneity in Acetonitrile–Water
Binary Mixtures
by using Polarity-Resolved Solvation Dynamics. ChemPhysChem.

[16] Shirzad K., Viney C. (2025). Revisiting time-dependent growth and nucleation rates in the Johnson–Mehl–Avrami–Kolmogorov
equation. Soc. Open Sci..

[17] Liu Z., Kalin M. L., Liu B., Cao S., Huang X. (2024). Kinetic network
models to elucidate aggregation dynamics of aggregation-induced emission
systems. Aggregate.

[18] Fernández E. J., Laguna A., López-De-Luzuriaga J. M., Monge M., Montiel M., Olmos M. E., Rodríguez-Castillo M. (2006). Photophysical
and Theoretical Studies on Luminescent Tetranuclear Coinage Metal
Building Blocks. Organometallics.

[19] Fernández E. J., Gimeno M. C., Laguna A., López-De-Luzuriaga J. M., Monge M., Pyykkö P., Sundholm D. (2000). Luminescent characterization
of solution oligomerization process mediated Gold–Gold interactions.
DFT calculations on [Au_2_Ag_2_R_4_L_2_]_n_ Moieties. J. Am. Chem.
Soc..

[20] De
Juan A., Jaumot J., Tauler R. (2014). Multivariate Curve Resolution (MCR).
Solving the mixture analysis problem. Anal.
Methods.

[21] De
Juan A., Tauler R. (2021). Multivariate Curve Resolution: 50 years addressing
the mixture analysis problem – A review. Anal. Chim. Acta.

[22] Garrido M., Rius F. X., Larrechi M. S. (2008). Multivariate curve
resolution–alternating
least squares (MCR-ALS). applied to spectroscopic data from monitoring
chemical reactions processes. Anal. Bioanal.
Chem..

[23] Williams G., Watts D. C. (1970). Non-symmetrical dielectric relaxation behaviour arising
from a simple empirical decay function. Trans.
Faraday Soc..

[24] Lukichev A. (2019). Lukichev,
Physical meaning of the stretched exponential Kohlrausch function. Phys. Lett. A.

[25] Boys S. F., Bernardi F. (1970). The calculation of
small molecular interactions by
the differences of separate total energies. Some procedures with reduced
errors. Mol. Phys..

[26] Klamt A., Schüürmann G. (1993). COSMO: A new
approach to dielectric
screening in solvents with explicit expressions for the screening
energy and its gradient. J. Chem. Soc., Perkin
Trans. 2.

[27] Savitzky A., Golay M. J. E. (1964). Smoothing and differentiation of data by simplified
least squares procedures. Anal. Chem..

[28] Cleveland W. S. (1979). Robust
locally weighted regression and smoothing scatterplots. J. Am. Stat. Assoc..

[29] Becke A. D. (1988). Density-functional
exchange-energy approximation with correct asymptotic behavior. Phys. Rev. A.

[30] Perdew J. P. (1986). Density-functional
approximation for the correlation energy of the inhomogeneous electron
gas. Phys. Rev. B.

[31] Weigend F., Ahlrichs R. (2005). Balanced basis sets
of split valence, triple zeta valence
and quadruple zeta valence quality for H to Rn: design and assessment
of accuracy. Phys. Chem. Chem. Phys..

[32] Grimme S., Ehrlich S., Goerigk L. (2011). Effect of
the damping function in
dispersion corrected density functional theory. J. Comput. Chem..

[33] Eichkorn K., Treutler O., Öhm H., Häser M., Ahlrichs R. (1995). Auxiliary basis sets to approximate Coulomb potentials. Chem. Phys. Lett..

[34] TURBOMOLE V8.0 2025, a Development Of University Of Karlsruhe And Forschungszentrum Karlsruhe GmbH, 1989–2007; TURBOMOLE GmbH, 2007. https://www.turbomole.org.

[35] Franzke Y. J., Holzer C., Andersen J. H., Begušić T., Bruder F., Coriani S., Della Sala F., Fabiano E., Fedotov D. A., Fürst S., Gillhuber S., Grotjahn R., Kaupp M., Kehry M., Krstić M., Mack F., Majumdar S., Nguyen B. D., Parker S. M., Pauly F., Pausch A., Perlt E., Phun G. S., Rajabi A., Rappoport D., Samal B., Schrader T., Sharma M., Tapavicza E., Treß R. S., Voora V., Wodyński A., Yu J. M., Zerulla B., Furche F., Hättig C., Sierka M., Tew D. P., Weigend F. (2023). TURBOMOLE: Today and
tomorrow. J. Chem. Theory Comput..

[36] Schäfer A., Klamt A., Sattel D., Lohrenz J. C. W., Eckert F. (2000). COSMO Implementation
in TURBOMOLE: Extension of an efficient quantum chemical code towards
liquid systems. Phys. Chem. Chem. Phys..

