# Supplementary material for: Thermally Driven Reorganization of Aurophilic AIE Aggregates in Microheterogeneous Acetonitrile/Water Media
Source: Inorg Chem. 2026 Aug 20;65(38):22521–31. doi: 10.1021/acs.inorgchem.6c03111 (PMC13625962; doi:10.1021/acs.inorgchem.6c03111)
Supplement: Supplementary file 1 [file ic6c03111_si_001.pdf]

SUPPORTING INFORMATION

Thermally Driven Reorganization of Aurophilic AIE Aggregates in Microheterogeneous Acetonitrile/Water Media

Ainhoa Rodríguez-Gobernado,[a] Inés Soldevilla,[a] Andrea Pinto,[b,c] Isabel Esteban-Díez,[a] Laura Rodríguez,[b,c] Miguel Monge,[a] María Rodríguez-Castillo,[a] José María López-de-Luzuriaga*[a]*

[a] Departamento de Química, Instituto de Investigación en Química de la Universidad de La Rioja (IQUR), Universidad de La Rioja, Complejo Científico Tecnológico, 26006-Logroño, Spain.

[b] Departament de Química Inorgànica i Orgànica, Secció de Química Inorgànica, Universitat de Barcelona, Martí i Franquès 1−11, Barcelona E-08028, Spain

[c] Institut de Nanociència i Nanotecnologia. Universitat de Barcelona, Barcelona 08028, Spain

Email: maria.rodriguez@unirioja.es, josemaria.lopez@unirioja.es

Time-dependent emissions at 25 ºC

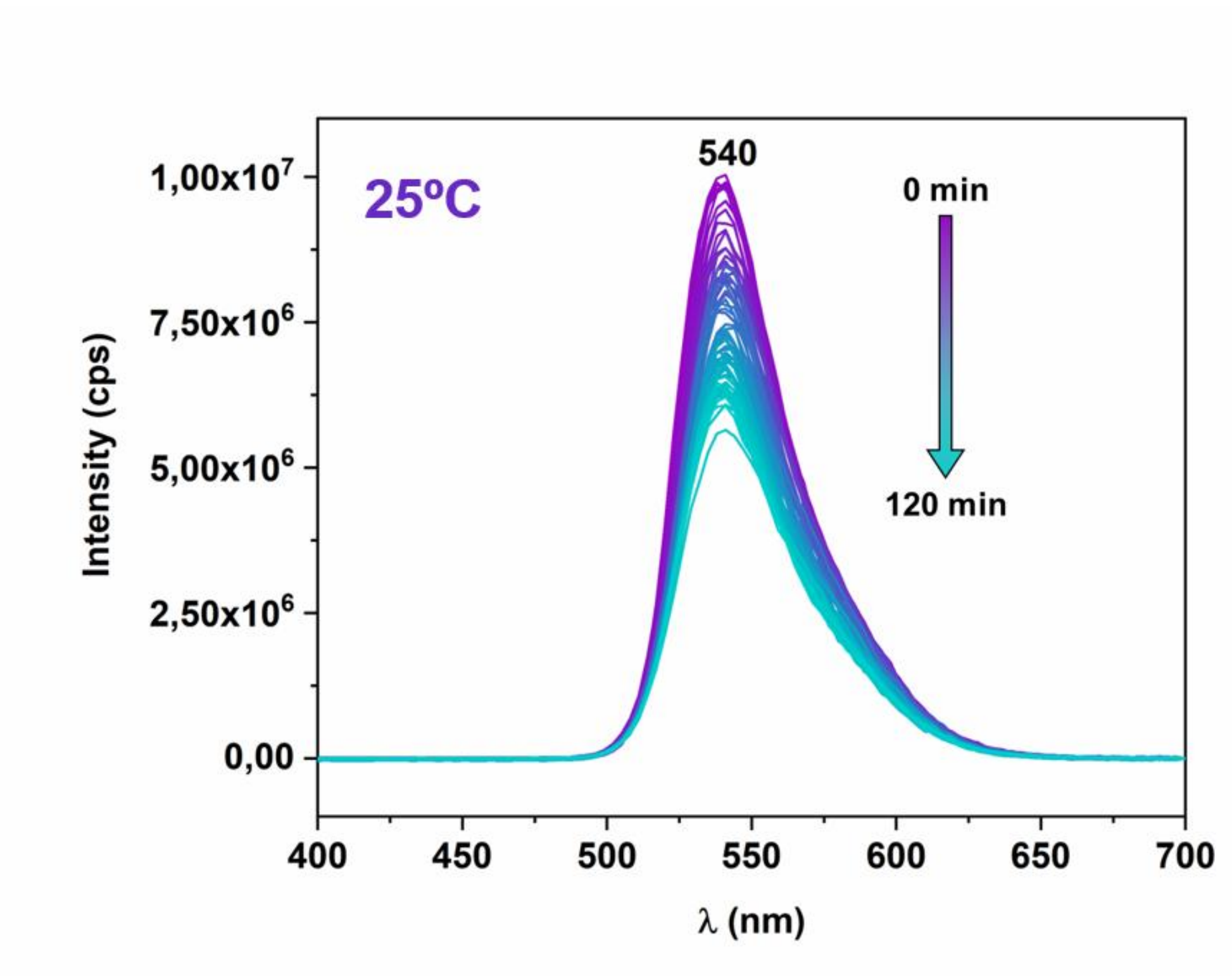

Figure S1. Emission spectra for complex $[Au_2Ag_2(C_6F_5)_4(N{\equiv}CCH_3)_2]_n$ in acetonitrile/water (20:80 v/v) at 1 x 10^{-4} M, during 120 min.

DLS results

Table S1. Intensity, Volume and Number-weighted DLS results for complex $[Au_2Ag_2(C_6F_5)_4(N{\equiv}CCH_3)_2]_n$ in acetonitrile/water (20:80 v/v) at a 1 x 10^{-4} M

TIME (min)	Intensity weighted (nm)	Volume weighted (nm)	Number weighted (nm)
0	1088, 65	1200, 65	57
2	5243, 469	5243, 573	424
4	1178	1157	1184
6	3001	3001	3001
8	10000, 1468	1468	1468
10	8236, 689	8236, 747	747
12	3293, 116	3293, 100	100
14	9391, 1836	8820, 1724	1724
16	9268, 1192	8590, 1192	1192
18	3153	3153	3153
20	2997	2993	2997
30	9380, 1896	9380, 1896	1896
35	5457	5457	5457

Table S2. Intensity-weighted DLS results for complex $[Au_2Ag_2(C_6F_5)_4(N{\equiv}CCH_3)_2]_n$ in acetonitrile/water (20:80 v/v) at a 3 x 10^{-5} M

TIME (min)	Intensity weighted (nm)	Volume weighted (nm)	Number weighted (nm)
0	886	886	886
2	8866, 438	7861, 557	557, 344
4	1968, 156	1968, 156	123
6	2590, 139	2590, 139	123
8	1076, 67	1227, 59	52
10	6003, 1095, 85	6003, 800, 72	72
12	8935, 535	7983, 535	535

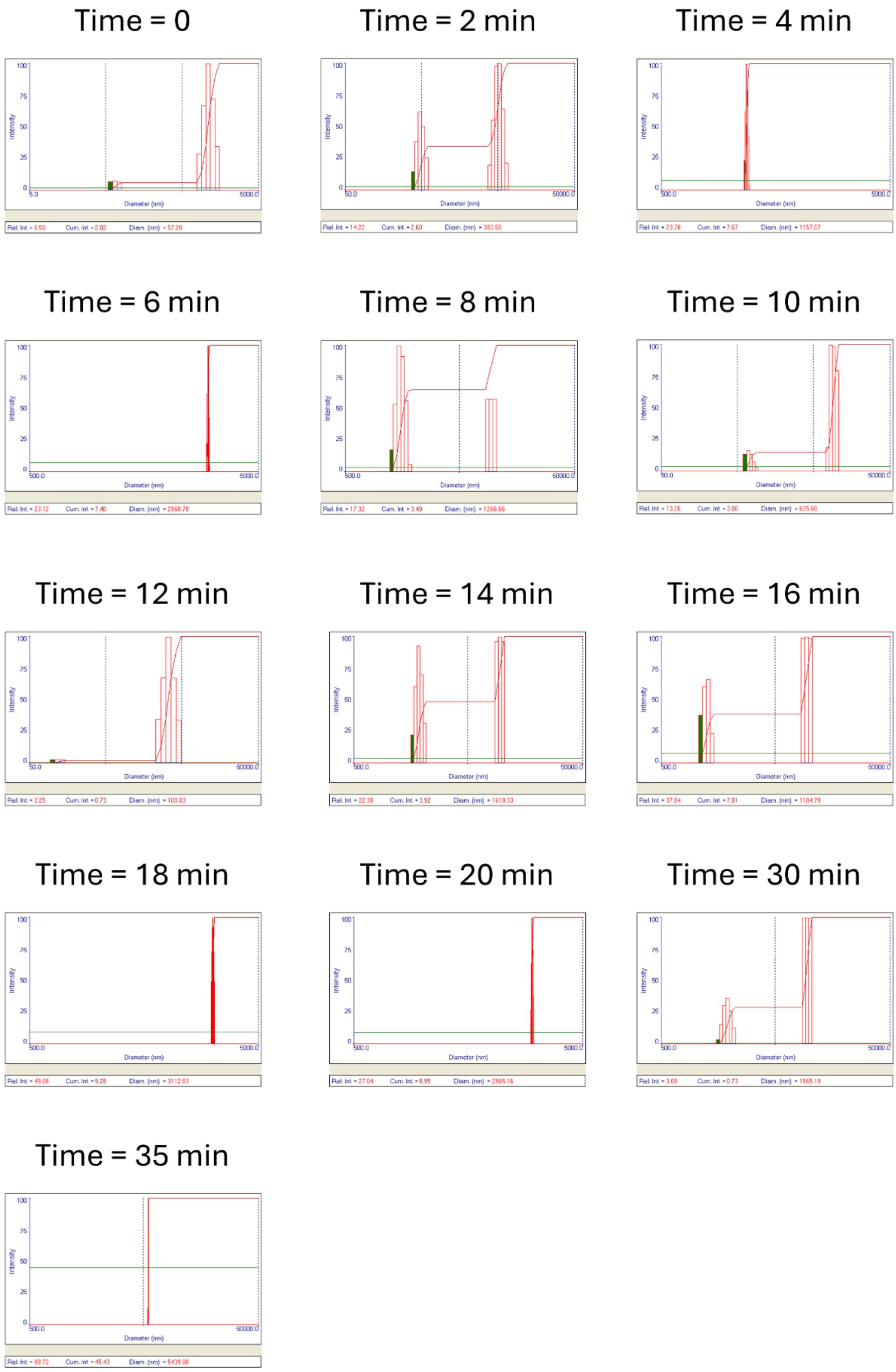

Figure S2. Intensity-weighted DLS graphical results for complex $[Au_2Ag_2(C_6F_5)_4(N{\equiv}CCH_3)_2]_n$ in acetonitrile/water (20:80 v/v) at 1 x 10^{-4} M

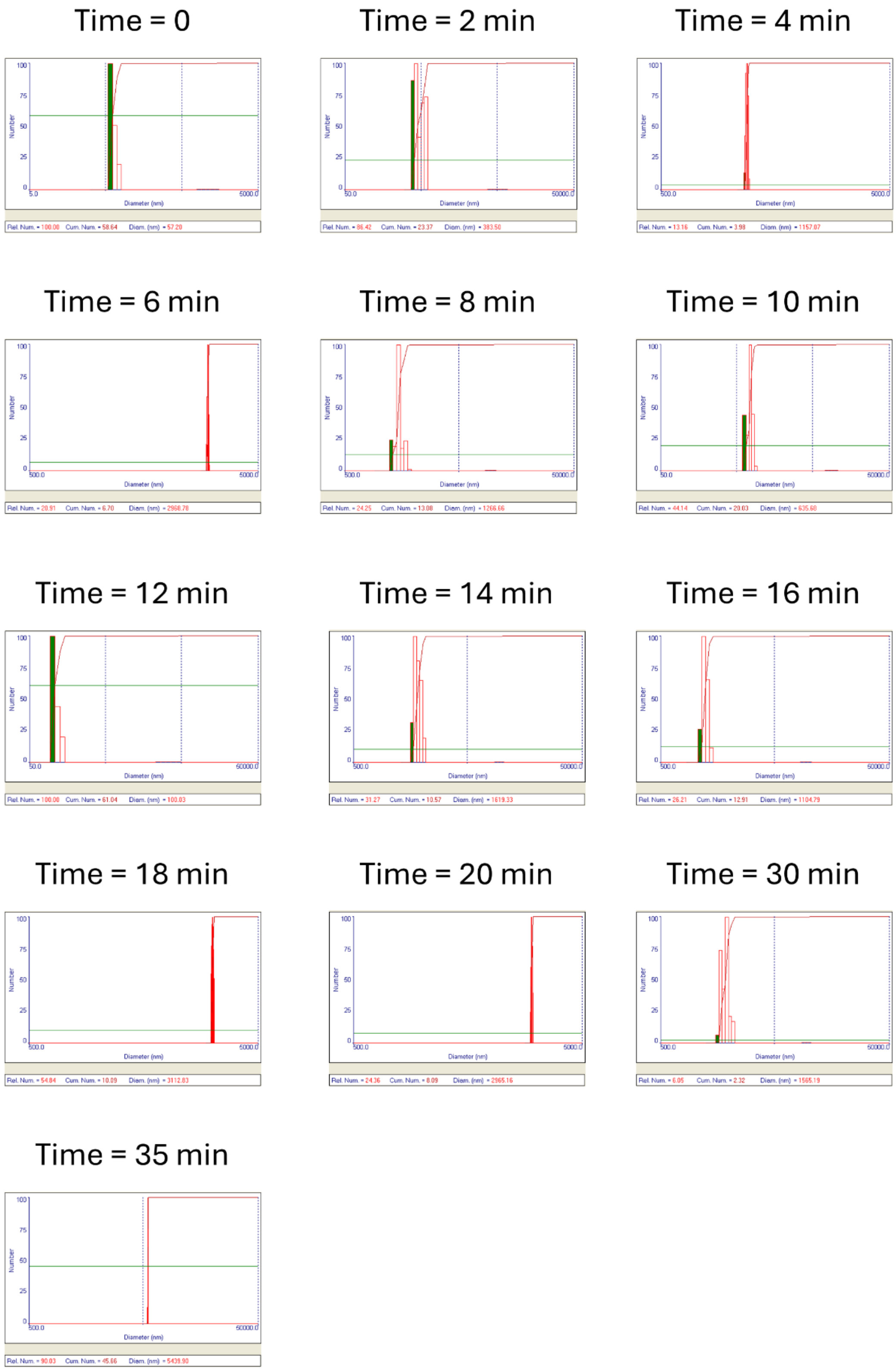

Figure S3. Number-weighted DLS graphical results for complex $[Au_2Ag_2(C_6F_5)_4(N{\equiv}CCH_3)_2]_n$ in acetonitrile/water (20:80 v/v) at 1 x 10^{-4} M

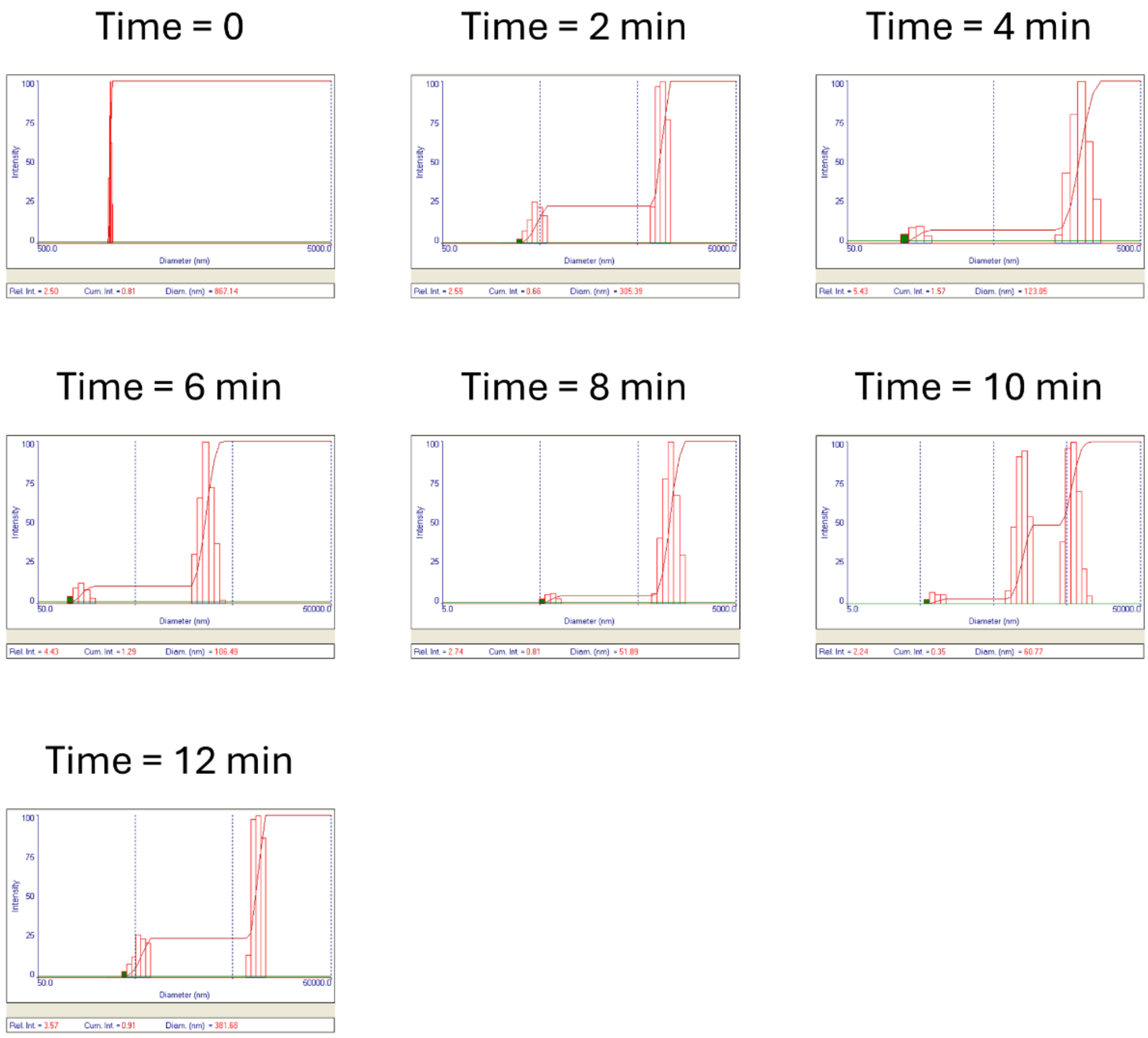

Figure S4. Intensity-weighted DLS graphical results for complex $[Au_2Ag_2(C_6F_5)_4(N{\equiv}CCH_3)_2]_n$ in acetonitrile/water (20:80 v/v) at 3 x 10^{-5} M.

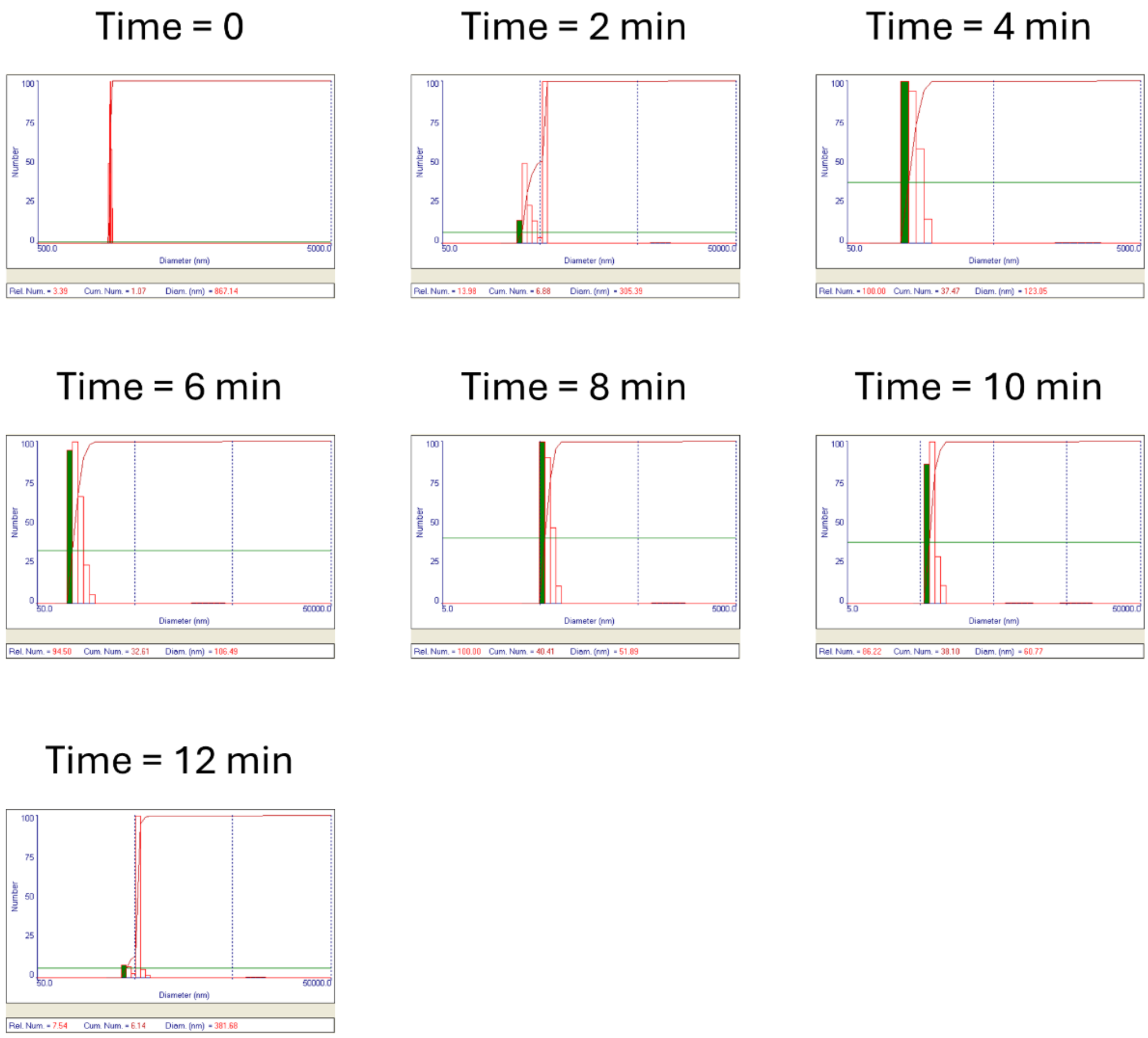

Figure S5. Number-weighted DLS graphical results for complex $[Au_2Ag_2(C_6F_5)_4(N{\equiv}CCH_3)_2]_n$ in acetonitrile/water (20:80 v/v) at 3 x 10^{-5} M.

Kinetic models employed

A set of eight kinetic models was evaluated In the multiparametric fitting procedure: single-exponential (monoexponential), offset-corrected monoexponential, lag-time monoexponential, Avrami (nucleation–growth), Kohlrausch–Williams–Watts (KWW), restricted biexponential, offset-corrected KWW, and Oosawa–Kasai models. These models span from simple first-order descriptions to more flexible empirical formulations and mechanistic nucleation–growth approaches, allowing for an unbiased evaluation of the kinetic behaviour.

- **MONO**: Monoexponential model (Single-exponential decay/growth model):

$$C(t) = C_0 + A\,e^{-kt}$$

- **OFFSET**: Monoexponential model with offset (Offset-corrected single-exponential model):

$$C(t) = C_0 + C_\infty\left(1 - e^{-kt}\right)$$

- **LAG**: Monoexponential model with lag time (Lag-time single-exponential model):

$$C(t) = \begin{cases} C_0 & t < t_{\text{lag}} \\ C_0 + A\left(1 - e^{-k(t-t_{\text{lag}})}\right) & t \geq t_{\text{lag}} \end{cases}$$

- **AVRAMI**: Avrami model (Avrami nucleation–growth model):

$$C(t) = C_0 + C_\infty\left(1 - e^{-(kt)^n}\right)$$

- **KWW**: KWW model (Kohlrausch–Williams–Watts model / stretched or compressed exponential):

$$C(t) = C_0 + A\left(1 - e^{-(kt)^\beta}\right)$$

- **BIEXP**: Restricted biexponential model (Biexponential model with constrained amplitudes):

$$C(t) = C_0 + A_1 e^{-k_1 t} + A_2 e^{-k_2 t}$$

- **KWW_OFFSET**: Offset-corrected KWW model (Offset-corrected Kohlrausch–Williams–Watts model / compressed-exponential with asymptote):

$$C(t) = C_0 + C_\infty \left(1 - e^{-(kt)^\beta}\right)$$

- **OK**: Oosawa–Kasai model (Oosawa–Kasai nucleation-elongation model):

$$C(t) = C_0 + f(t; k_{\text{act}}, k_{\text{elong}}, \dots)$$

$$C(t) = C_0 + C_\infty \left[1 - \exp\left(-k_{\text{elong}}\left(t - \frac{1 - e^{-k_{\text{act}} t}}{k_{\text{act}}}\right)\right)\right]$$

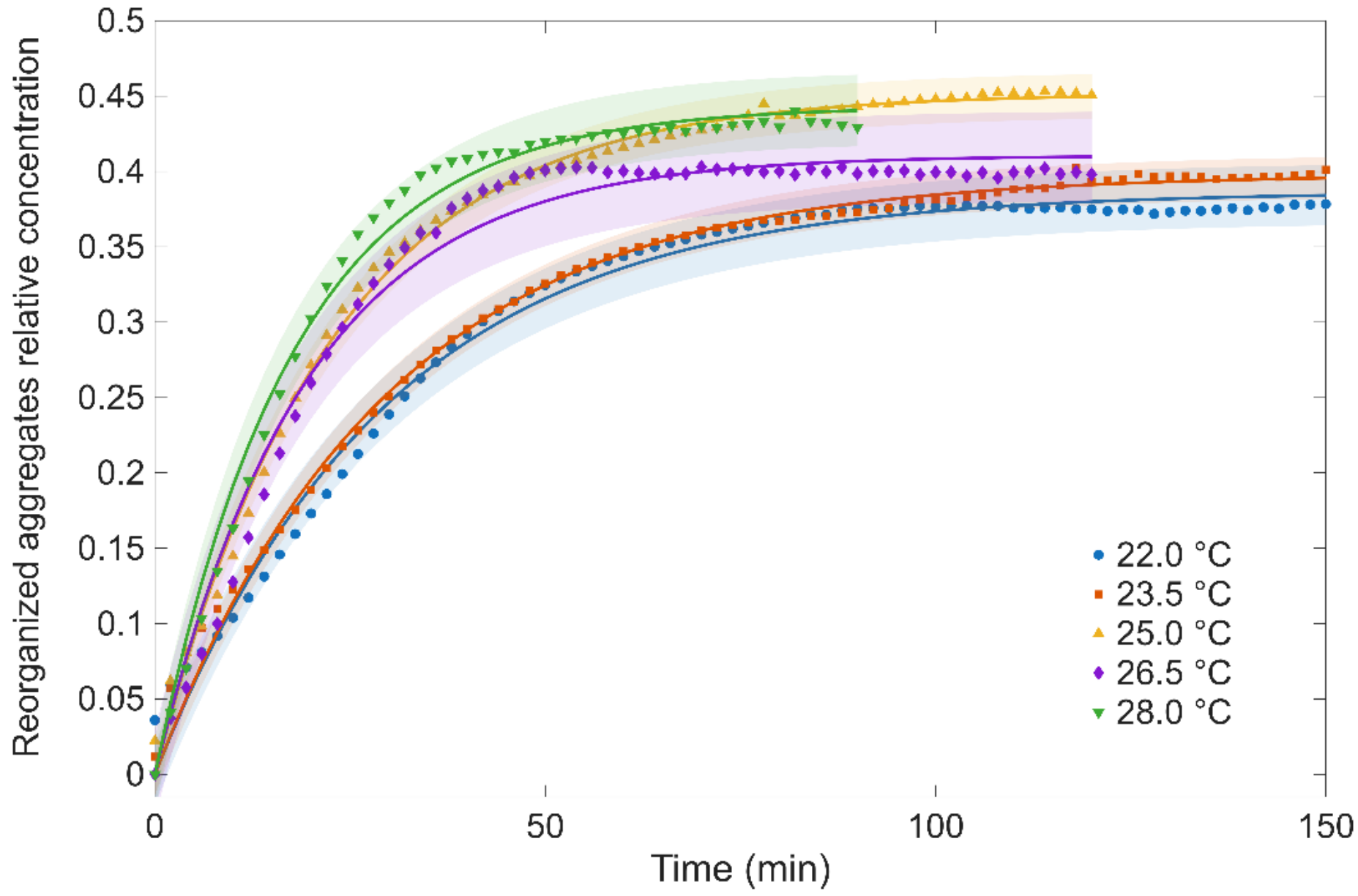

Figure S6. Concentration profiles of the reorganized aggregates and corresponding fits obtained using the **MONO**: Monoexponential model (Single-exponential decay/growth model). Symbols represent the MCR-ALS-resolved concentration profiles, solid lines the fitted profiles, and shaded regions the 95% confidence intervals.

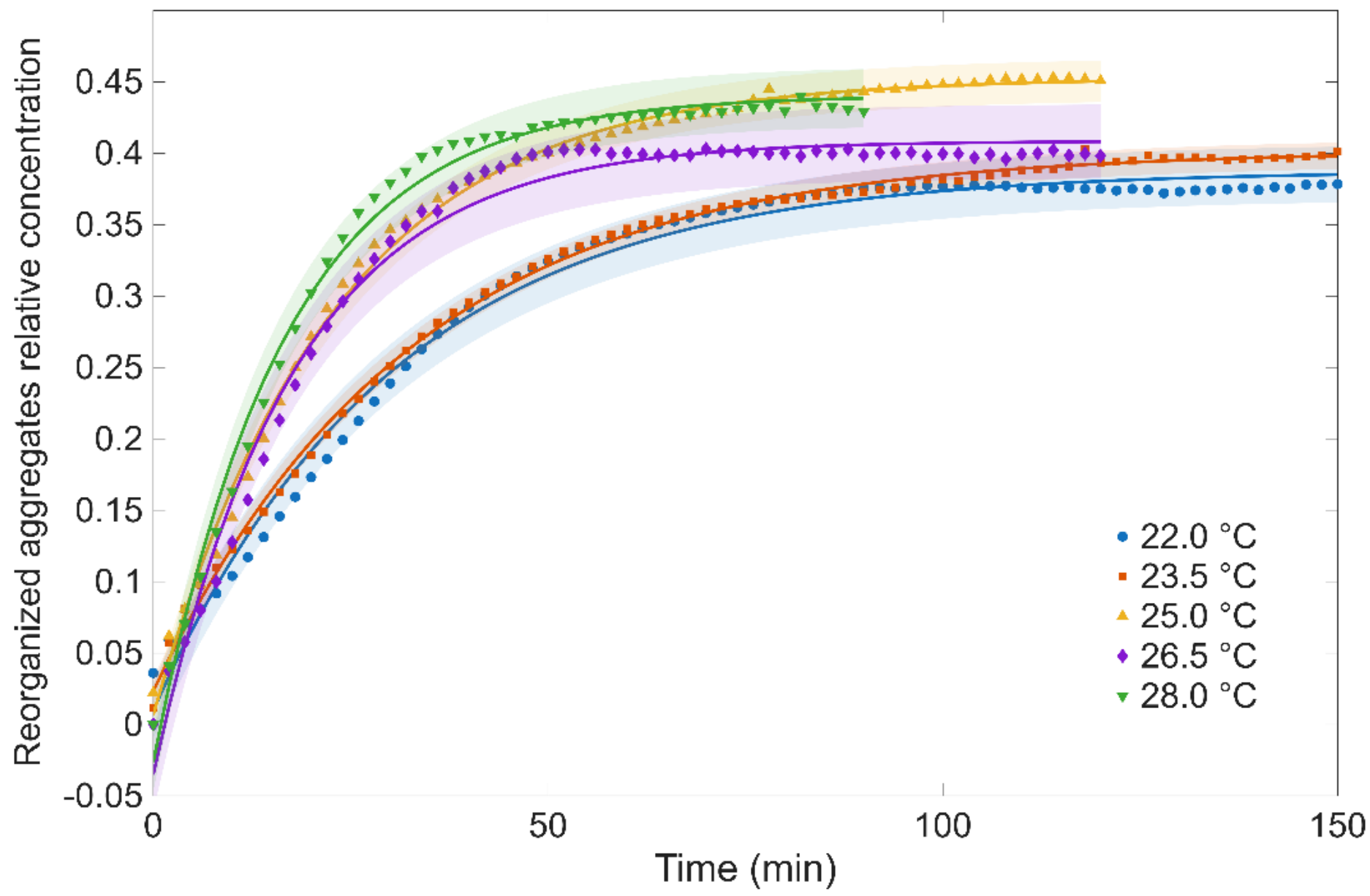

Figure S7. Concentration profiles of the reorganized aggregates and corresponding fits obtained using the **OFFSET**: Monoexponential model with offset (Offset-corrected single-exponential model). Symbols represent the MCR-ALS-resolved concentration profiles, solid lines the fitted profiles, and shaded regions the 95% confidence intervals.

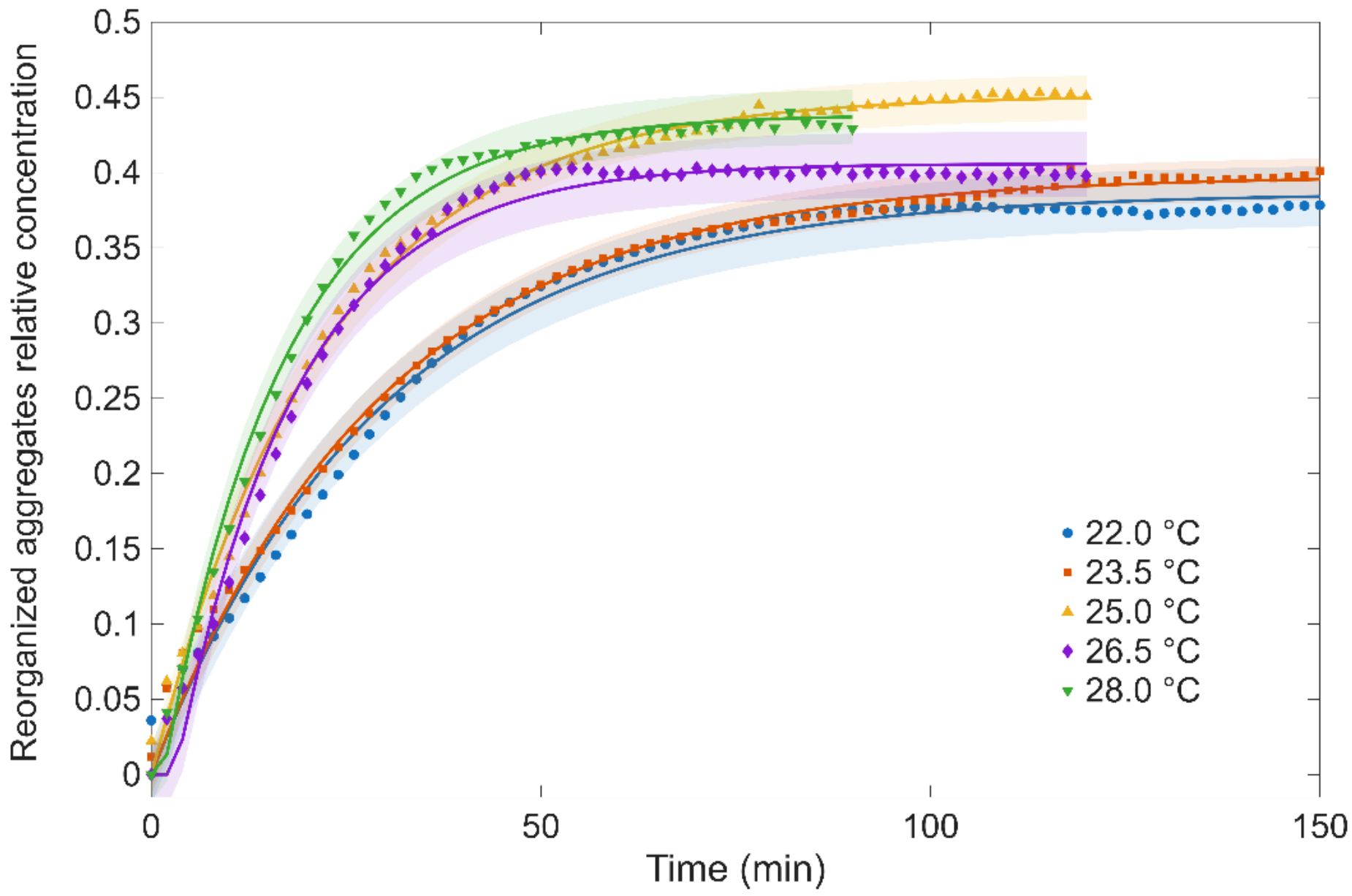

Figure S8. Concentration profiles of the reorganized aggregates and corresponding fits obtained using the **LAG**: Monoexponential model with lag time (Lag-time single-exponential model). Symbols represent the MCR-ALS-resolved concentration profiles, solid lines the fitted profiles, and shaded regions the 95% confidence intervals.

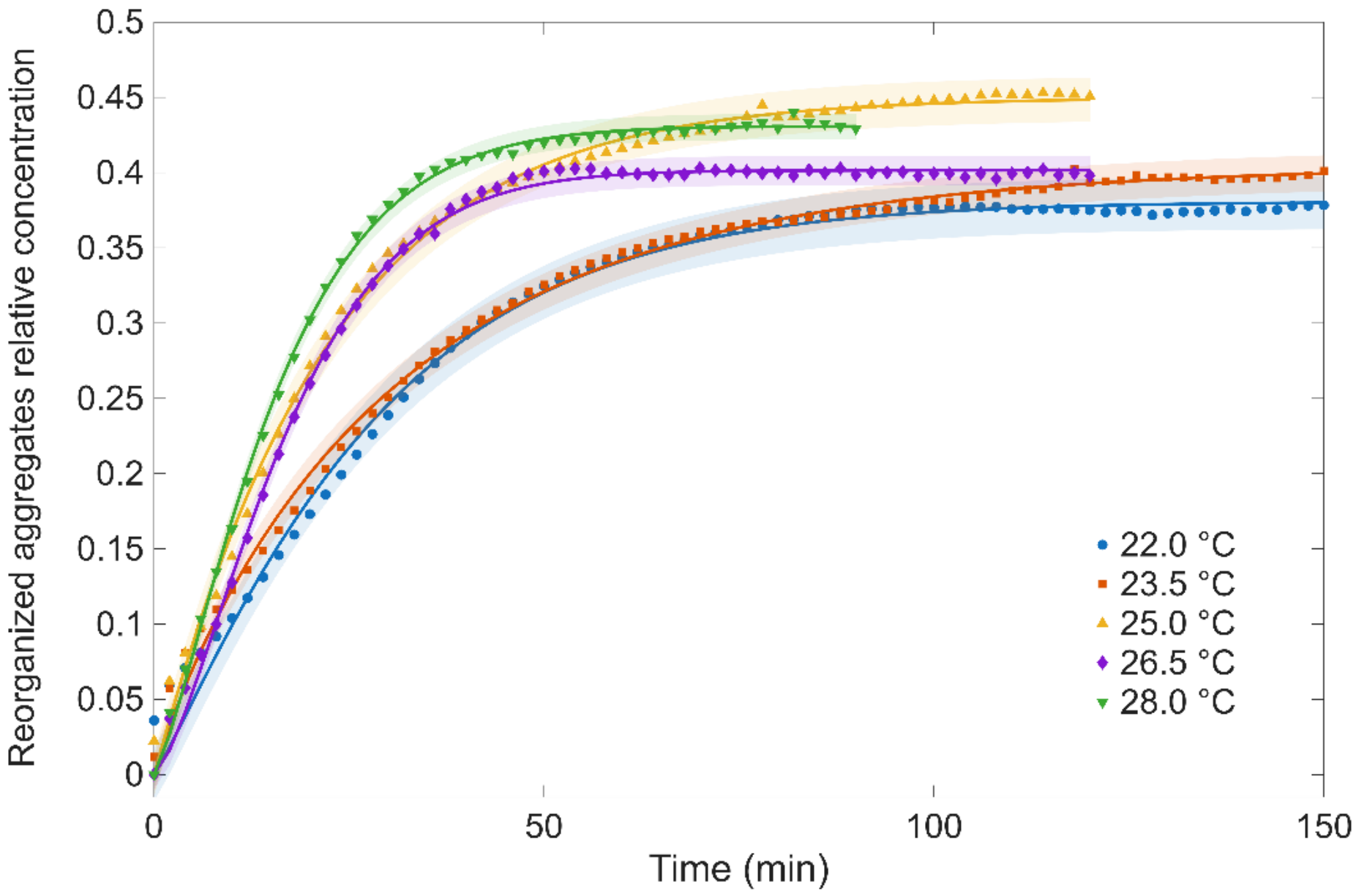

Figure S9. Concentration profiles of the reorganized aggregates and corresponding fits obtained using the **AVRAMI**: Avrami model (Avrami nucleation–growth model). Symbols represent the MCR-ALS-resolved concentration profiles, solid lines the fitted profiles, and shaded regions the 95% confidence intervals.

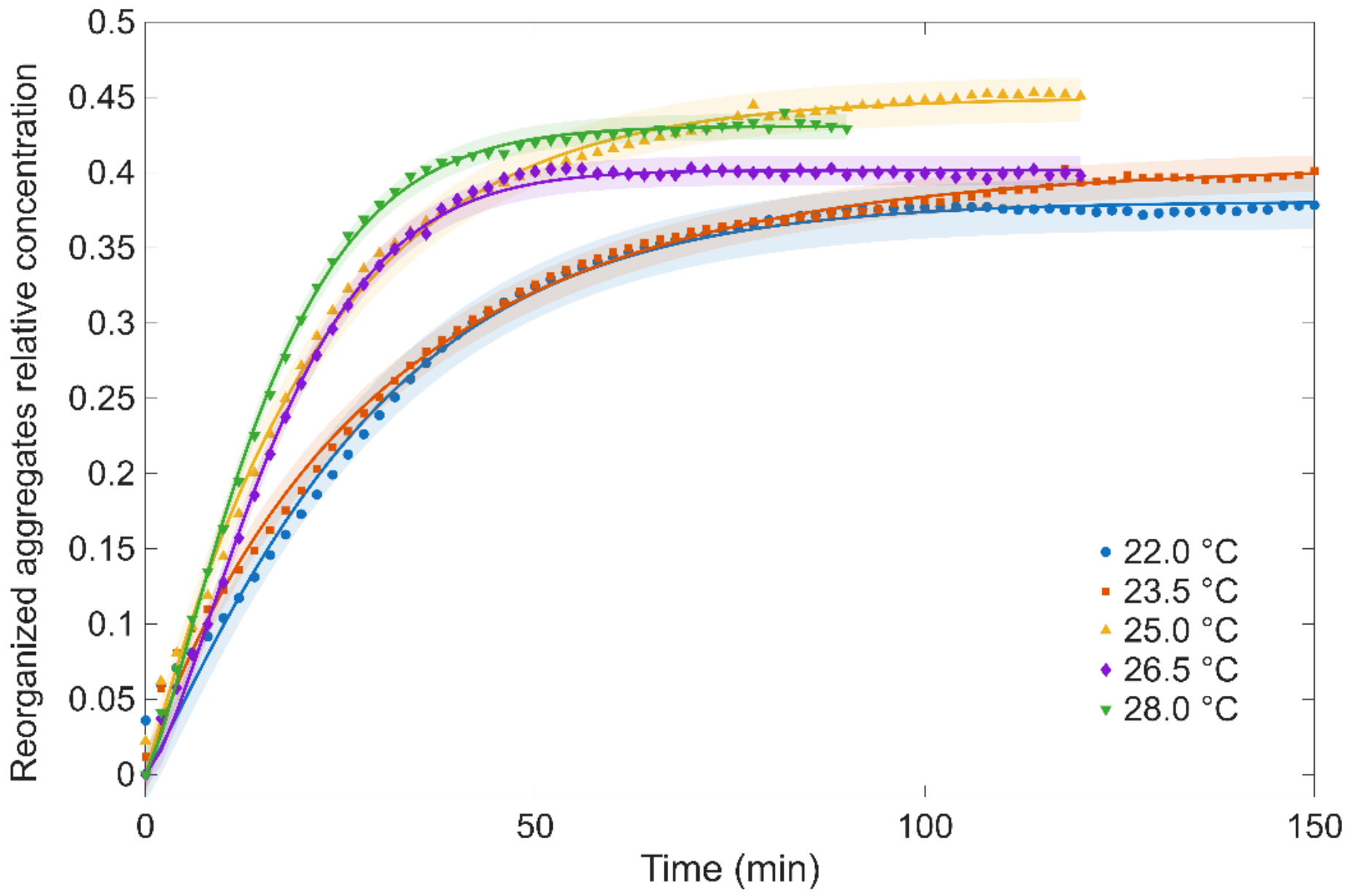

Figure S10. Concentration profiles of the reorganized aggregates and corresponding fits obtained using the **KWW** model (Kohlrausch–Williams–Watts model / stretched or compressed exponential). Symbols represent the MCR-ALS-resolved concentration profiles, solid lines the fitted profiles, and shaded regions the 95% confidence intervals.

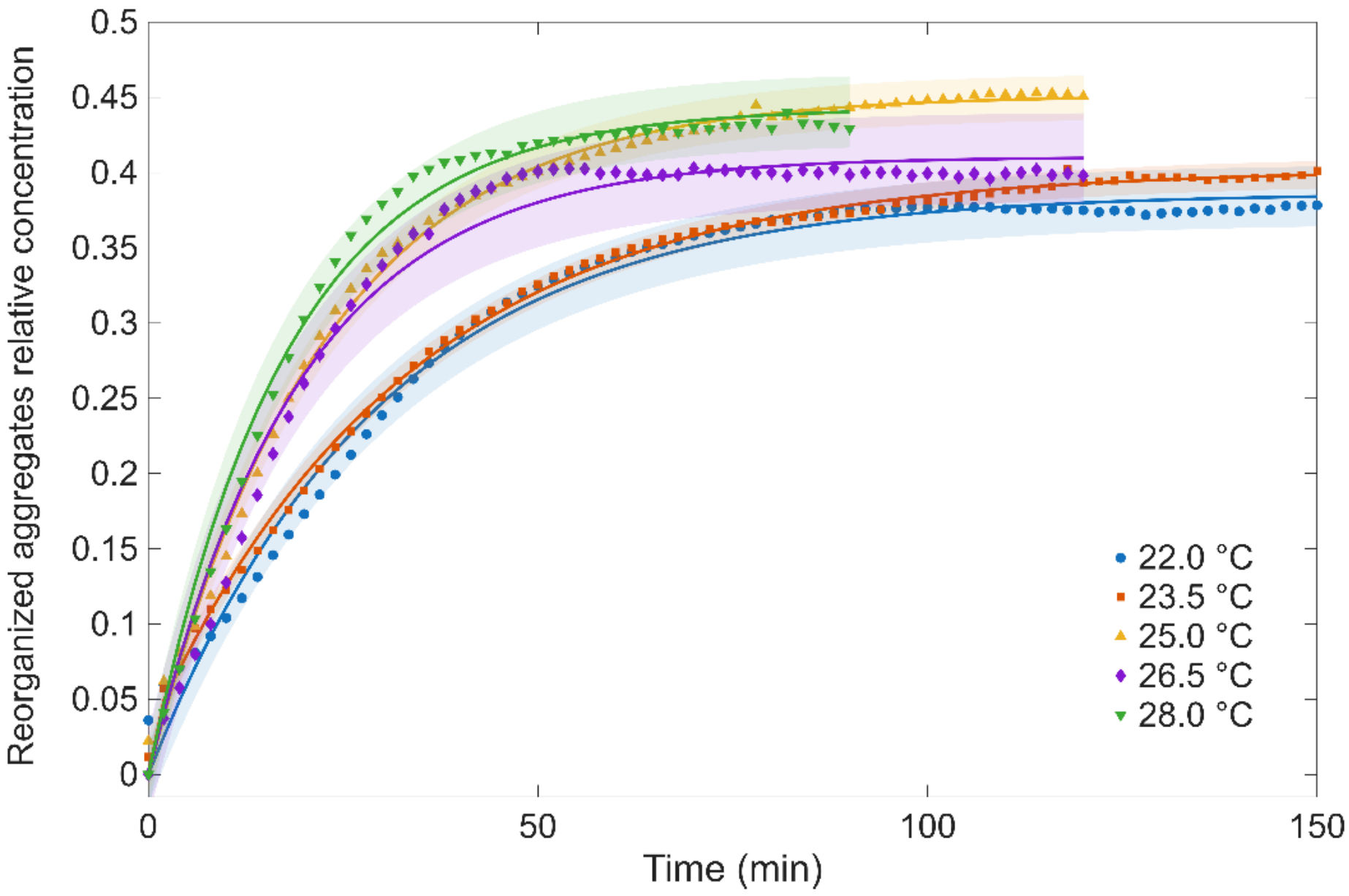

Figure S11. Concentration profiles of the reorganized aggregates and corresponding fits obtained using the **BIEXP**: Restricted biexponential model (Biexponential model with constrained amplitudes). Symbols represent the MCR-ALS-resolved concentration profiles, solid lines the fitted profiles, and shaded regions the 95% confidence intervals.

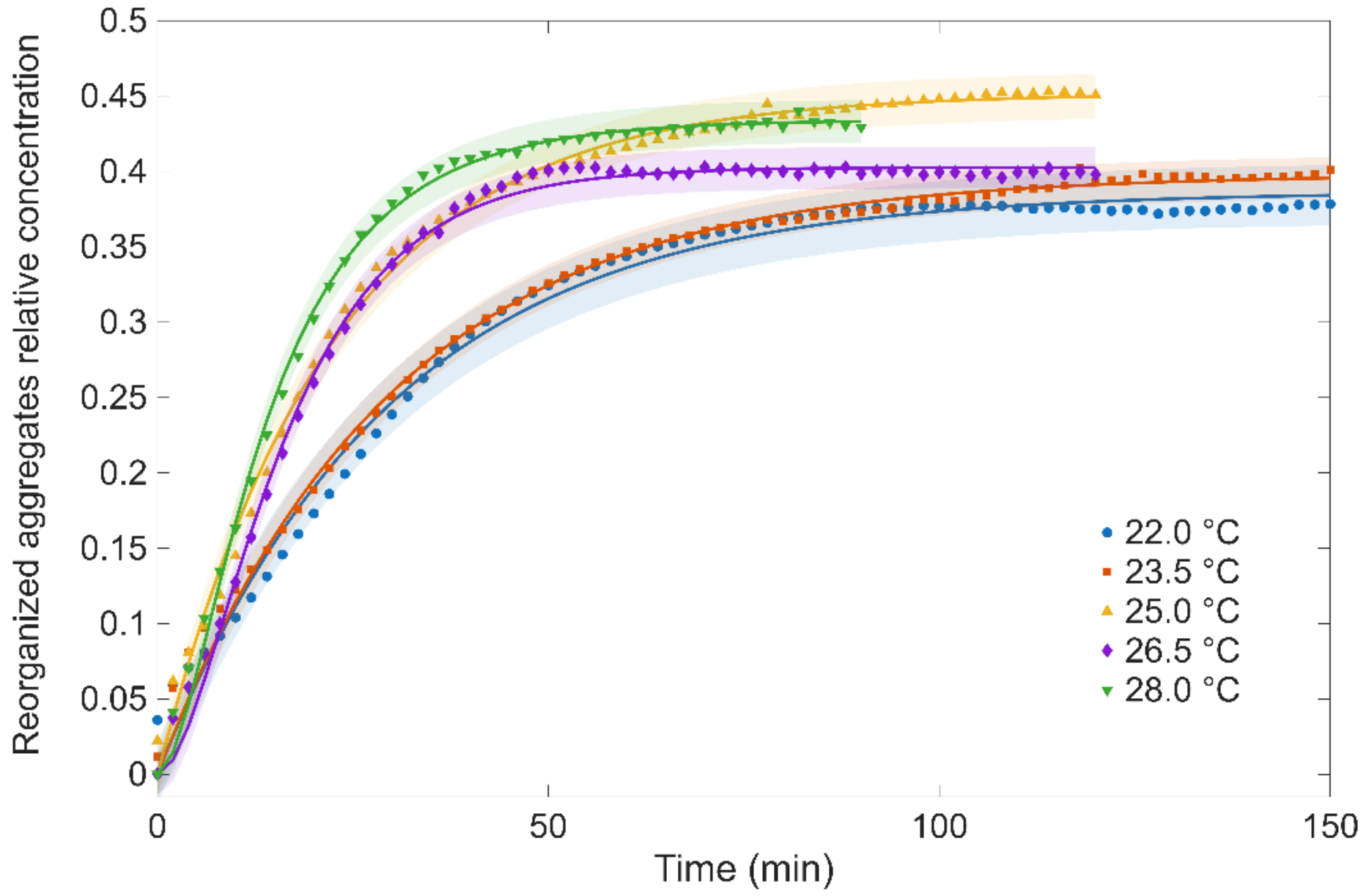

Figure S12. Concentration profiles of the reorganized aggregates and corresponding fits obtained using the **OK**: Oosawa–Kasai model (Oosawa–Kasai nucleation-elongation model). Symbols represent the MCR-ALS-resolved concentration profiles, solid lines the fitted profiles, and shaded regions the 95% confidence intervals.

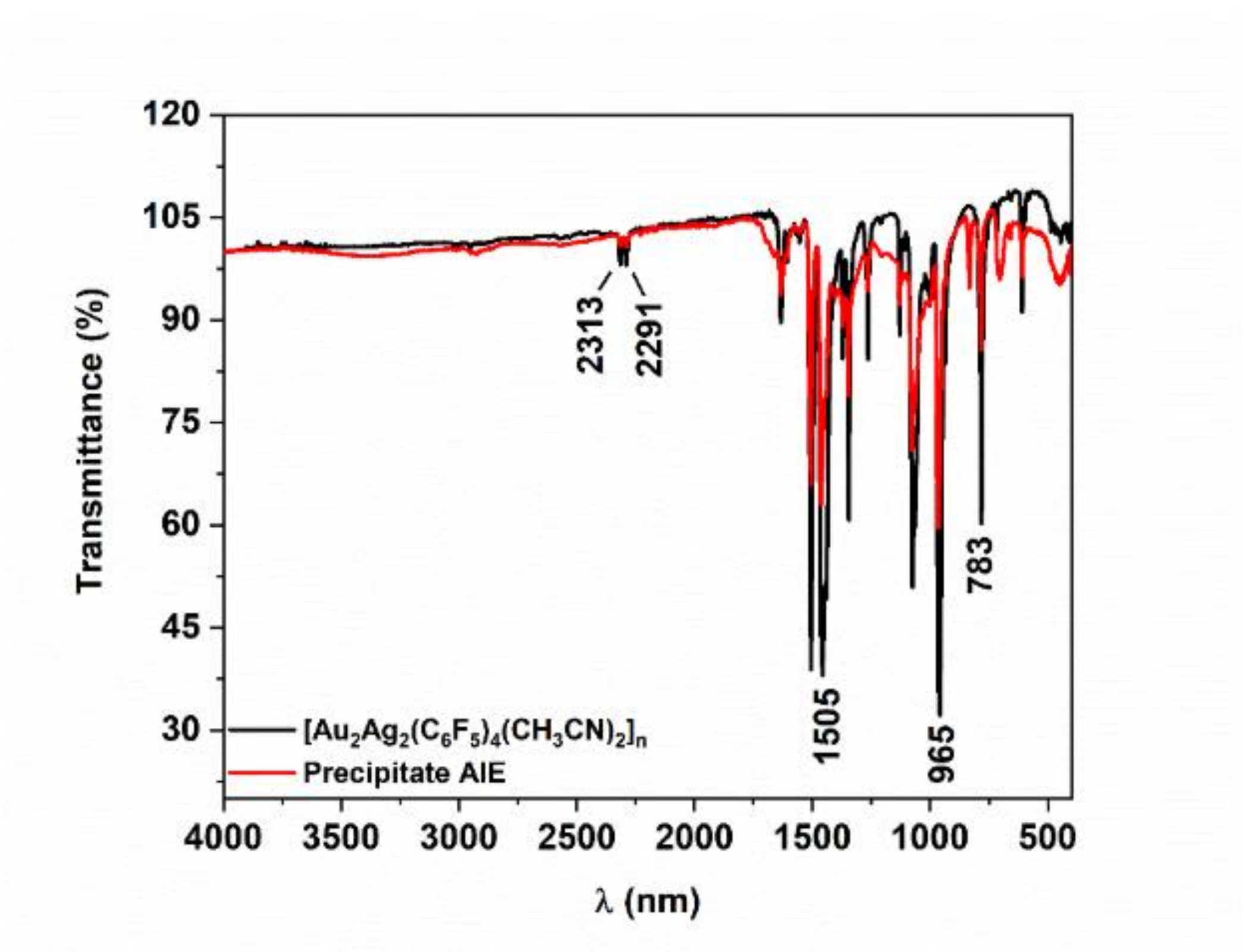

Figure S13. FTIR spectra of complex $[Au_2Ag_2(C_6F_5)_4(N{\equiv}CCH_3)_2]_n$ and of obtained solid after the aggregation process in solid state.

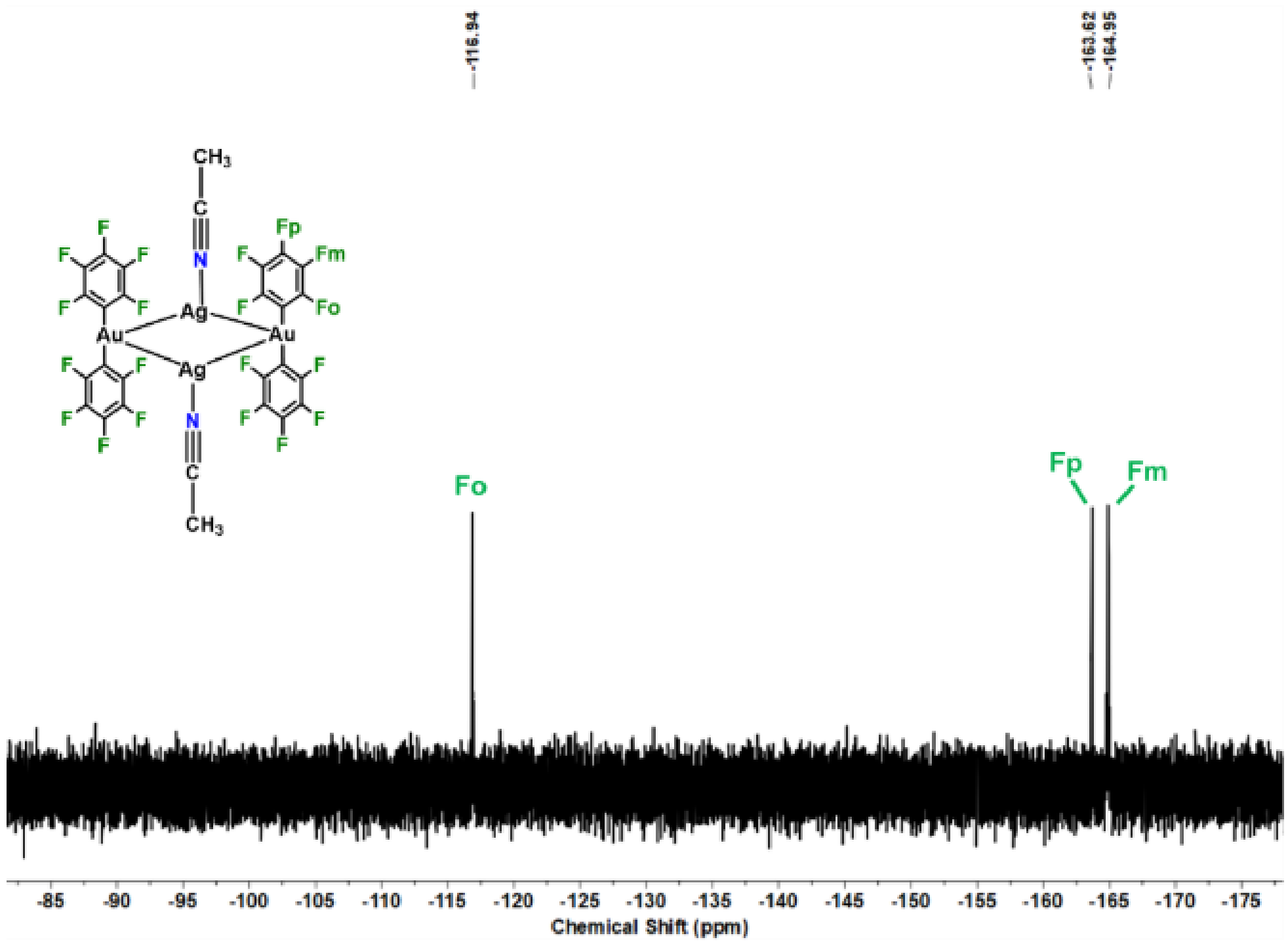

Figure S14. ^{19}F NMR spectra of the obtained solid after the aggregation process in D3-acetonitrile solution.
